# Combining global land cover datasets to quantify agricultural expansion into forests in Latin America: Limitations and challenges

**DOI:** 10.1371/journal.pone.0181202

**Published:** 2017-07-13

**Authors:** Florence Pendrill, U. Martin Persson

**Affiliations:** Division of Physical Resource Theory, Department of Space, Earth & Environment, Chalmers University of Technology, Göteborg, Sweden; University of Maryland at College Park, UNITED STATES

## Abstract

While we know that deforestation in the tropics is increasingly driven by commercial agriculture, most tropical countries still lack recent and spatially-explicit assessments of the relative importance of pasture and cropland expansion in causing forest loss. Here we present a spatially explicit quantification of the extent to which cultivated land and grassland expanded at the expense of forests across Latin America in 2001–2011, by combining two “state-of-the-art” global datasets (Global Forest Change forest loss and GlobeLand30-2010 land cover). We further evaluate some of the limitations and challenges in doing this. We find that this approach does capture some of the major patterns of land cover following deforestation, with GlobeLand30-2010’s Grassland class (which we interpret as pasture) being the most common land cover replacing forests across Latin America. However, our analysis also reveals some major limitations to combining these land cover datasets for quantifying pasture and cropland expansion into forest. First, a simple one-to-one translation between GlobeLand30-2010’s Cultivated land and Grassland classes into cropland and pasture respectively, should not be made without caution, as GlobeLand30-2010 defines its Cultivated land to include some pastures. Comparisons with the TerraClass dataset over the Brazilian Amazon and with previous literature indicates that Cultivated land in GlobeLand30-2010 includes notable amounts of pasture and other vegetation (e.g. in Paraguay and the Brazilian Amazon). This further suggests that the approach taken here generally leads to an underestimation (of up to ~60%) of the role of pasture in replacing forest. Second, a large share (~33%) of the Global Forest Change forest loss is found to still be forest according to GlobeLand30-2010 and our analysis suggests that the accuracy of the combined datasets, especially for areas with heterogeneous land cover and/or small-scale forest loss, is still too poor for deriving accurate quantifications of land cover following forest loss.

## 1 Introduction

Deforestation in the tropics has a large impact on biodiversity, ecosystem services, and carbon emissions. One of the major causes of deforestation in the tropics is expansion of cropland and pastures associated with rising demand for agricultural products [1,2], such as soybeans and beef [3]. This demand is increasingly driven by trade on global markets [4,5], creating distances between the consumption of agricultural commodities and the impacts of their production. The distant interactions, or teleconnections, between agricultural commodity demand and forest loss give rise to complex challenges in addressing deforestation, as interventions in one location may have unintended consequences elsewhere, for example through leakage or ‘indirect land-use change’ [6,7].

Attempts to counteract deforestation has led to a recent flurry of initiatives that seek to complement supply-side measures for tropical forest conservation, such as REDD+ (Reducing Emissions from Deforestation and Forest Degradation in Developing Countries), with demand-side measures, such as improved supply-chain sustainability through zero-deforestation commitments and commodity moratoria [8,9]. Such policies and interventions rely on adequate information on the contribution of different agricultural commodities to deforestation. In response to this, sub-national level data on trade of agricultural commodities are becoming available through a wealth of transparency initiatives [10]. These interventions could be further supported by spatially-explicit data on where pastures and cropland are expanding into forests, as these provide possibilities for linking sub-national environmental impacts to analyses at the global scale [11,12].

While some countries (most notably Brazil) have made use of remote sensing monitoring to tackle deforestation [13], most tropical countries still lack recent and spatially-explicit estimates of the proximate causes of deforestation [5,14–16]. However, while data on land use are still limited [17], an increased availability of remotely-sensed data, together with improved processing capabilities, has in recent years led to the publication of new global and regional datasets on land cover and forest loss at significantly higher spatial resolution than before (c. 30 m, rather than 0.3–1 km and up) for as recently as 2010 [18–21]. These datasets provide a comparatively quick way to gain detailed information on land cover and reveal patterns to forest cover change.

In this study, we seek to quantify where in Latin America pasture and cropland have expanded at the expense of forests. Additionally, we seek to evaluate to what extent freely-available global land use/land cover maps can be used for this purpose. As noted above, global and spatially-explicit data on land use (e.g. pasture and cropland) are limited, so instead we explore if currently available global datasets and maps on land cover and land cover change can be used to quantify where cropland and pasture expansion have replaced forest. Combining two “state-of-the-art” global datasets—Global Forest Change annual tree cover loss [19] followed by GlobeLand30-2010 land cover [21]—we carry out an assessment of land cover transitions (from forest to cultivated land, grassland or other land cover types), quantifying “post-forest loss land cover”. This is done at approximately 30-m resolution for the whole of Latin America, exceeding the spatial resolution and/or coverage of previous continental-scale studies of land-cover change in the region (e.g. [22–24]). We evaluate the results using additional, well-established datasets over the Brazilian Legal Amazon (PRODES and TerraClass) and published literature for other regions, allowing us to provide an assessment of how well the datasets used agree with more specific regional datasets. This is especially valuable for GlobeLand30-2010, which is a comparatively new land cover dataset (released in 2014) and that so far has only been subject to a few independent validations [21,25–27], none of which have been specific to Latin America. There is thus still room for improving the understanding of how GlobeLand30-2010 performs in different regions [25]. The comparison with the Brazilian datasets and with the previous literature also helps identify what type of information can be garnered from currently available global datasets, and reveals some limitations and remaining challenges in global land use / land cover mapping.

## 2 Methods and data

### 2.1 Study area

Latin America is home to about half of the world’s remaining tropical forests, with major biomes including the Amazon, along with drier forests/woodlands and savannahs, such as the Gran Chaco (spanning parts of Argentina, Bolivia, Brazil, and Paraguay), the Cerrado (in Brazil), and the Chiquitano (in Bolivia and Brazil). These forests not only store close to half of the biomass carbon of all tropical forests [28], but many have also been identified as prioritized areas for global biodiversity conservation [29].

Latin America also has some of the highest deforestation rates in the world [19,22], much of which is driven by large-scale commercial agriculture, producing both for domestic and international markets [4,5]. During the time period considered in this study, 2001–2011, more than half of the forest loss in Latin America occurred in the Brazilian Legal Amazon (BLA) [19,30].

### 2.2 Method overview

This study has two interconnected aims: (1) to quantify where pasture and cropland have expanded at the expense of forests in Latin America, and (2) to evaluate how well currently-available spatially-explicit, global datasets on land cover can be used for this purpose.

For the first aim, we quantify the relative contribution of pasture and cropland in replacing forests by combining spatially-explicit data on forest loss and on land cover, and use their respective timing to determine the subsequent land cover type for each deforested pixel. Henceforth, we use the term “post-loss land cover/use” to refer to the land cover/use classification of areas that were previously deforested. The post-loss land cover/uses can be used to gain an indication of which land uses (such as cropland and/or pasture) are proximate causes of forest loss (as is done by e.g. [23,31,32]).

However, the causes of forest loss are often complex and can be examined in different ways. Often a distinction is made between proximate causes and underlying drivers [33]. Proximate causes can be defined as *“a factor which intervenes close to the end of the causal chain”* [34], and are in turn a consequence of factors earlier in the causal chain, termed underlying (or indirect) causes or drivers. Common examples of underlying causes are changes to population, technology, culture, and/or institutions, along with predisposing environmental factors, which together influence the demand for different agricultural commodities, land, labour, and the cost of intensification versus extensification [6,33–35].

What we consider here is primarily relevant for the proximate causes of forest loss in the form of agricultural expansion (which is the dominant proximate cause of deforestation in Latin America [14,36]). Specifically, we seek to subdivide agricultural expansion into different types (cropland and pasture), by examining land cover following forest loss. An underlying assumption for quantifying the proximate causes of forest loss based on the analysis in this study is therefore that the land cover or land use that replaces forest reflects the proximate cause of that forest loss. This is a simplification, entailing for example that deforestation primarily for timber harvesting is not directly dealt with, nor are other causes unnoticeable from the changed land cover (such as storm damage or other natural forest loss, or clearing solely to claim property rights/tenure).

For the second aim, to evaluate the limitations and possibilities of using global datasets for assessing where cropland and pasture have expanded into forests, we examine the results of the post-loss analysis (and the constituent datasets) by comparing them to previous studies of proximate causes of deforestation in the literature, as well as to higher accuracy regional datasets for the Brazilian Legal Amazon.

### 2.3 Data sources

In choosing datasets, we compiled information on a number of freely available global datasets relating to forest loss and land cover (and use), using recent review articles [16,18,20,37], complemented by searches on Google Scholar. Datasets that were considered are summarised in S1 Table. These datasets were evaluated on the basis of their spatial and temporal coverage and resolution, the suitability of the classification for the intended purpose, and data quality as reported in accuracy assessments.

Based on this overview, we selected two global datasets for the main assessment: the Global Forest Change (GFC) annual tree cover loss dataset created by Hansen et al. [19] and GlobeLand30-2010, which is a land cover dataset provided by the National Geomatics Center of China (NGCC) [21]. The reason for this choice was that these datasets provide the highest available spatial resolution (c. 30 m) amongst currently available global datasets, while retaining overall accuracies over 80% [16,19–21]. The data are also relatively recent: GFC provides annual data 2001–2014, and GlobeLand30-2010 is for a single year around 2010, permitting attribution of post-forest loss land cover for the first decade of the 21^st^ century. Furthermore, GlobeLand30-2010 and GFC are both based primarily on Landsat data and provided at similar resolution, thus avoiding the need to compare and aggregate land cover classes at different pixel sizes during resampling (which can otherwise be challenging, as e.g. the type of land cover classes that can be detected at 30 m resolution differ conceptually from those that can be detected at 1 km [38]). A visual inspection of the datasets showed that they were generally well aligned.

The GFC annual tree cover loss data were used to identify where and when tree cover loss occurred. In this dataset, forests are defined by their physical attributes (Table 1), rather than by their function or the land use, and therefore not only include losses of natural/primary forest, but also, for example, harvesting of commercial forestry and shifting cultivation [19,39]. In this study we only consider gross forest loss, and do not account for any subsequent forest gain.

**Table 1:** Definitions for the key land cover / land use (change) classes in the four datasets used.

	Dataset			
Class	GFC	GlobeLand30-2010	PRODES	TerraClass
Forest	Trees or other vegetation exceeding a height of 5 m, and > 30% canopy cover (prior to loss) [39]	*“Land covered with trees*, *with vegetation cover over 30%[…]*, *and sparse woodland with cover 10–30%”* [40]	Primary/unchanged or slightly altered forest vegetation with continuous canopy (>10%, though primarily 70–100%) composed of native species [41]	
Forest loss (GFC) / Deforestation (P)	*“a ‘stand-replacement disturbance’*, *meaning the removal or mortality of all tree cover in a Landsat pixel”* [39]	n.a.	“*Areas recently deforested covered by soil*, *shrubs*, *herbage and felled trees with no defined land use at this stage”* [42]	n.a.
Grassland (GL) / Pasture (TC)	n.a.	“*Lands covered by natural grass with cover over 10%*, *etc*.*”* [40]	n.a.	“*Pasture in productive process […]”*. Divided into four sub-classes [42]
Cultivated land (GL) / Annual crops (TC)	n.a.	*“Land used for agriculture*, *horticulture and gardens*, *including paddy fields*, *irrigated and dry farmland*, *vegetation and fruit gardens*, *etc*.*”* [40] *“[…] irrigated farmlands*, *paddy fields*, *green houses cultivated land*, *artificial tame pastures*, *economic cultivated land (such as grape*, *coffee*, *and palm)*, *and abandoned arable lands*” [21]	n.a.	“*Extensive areas with predominance of annual crops[…]”* [42]

Dataset abbreviations: Global Forest Change (GFC), GlobeLand30-2010 (GL), PRODES (P) and TerraClass (TC). One assumption tested in the post-loss analysis is whether the GlobeLand30-2010 classes ‘Cultivated land’ and ‘Grassland’ can approximate forest loss for cropland and pasture, respectively.

GlobeLand30-2010 is produced using a combination of pixel- and object-based classification, along with manual verification, and its overall accuracy is estimated to approximately 80% and up ([21,25–27,43], summarised in Table A in S1 Appendix). It distinguishes between 10 land cover classes, including classes for Grassland, Cultivated land and Forest. For the purpose of quantifying to what extent pasture and cropland follow deforestation, a land use dataset might have been more suitable than a land cover dataset, with an obvious limitation with GlobeLand30-2010 being the lack of a pasture class. While there exists some global datasets on land use, crops, and livestock/cattle/grazing land, these are typically of significantly lower spatial resolution (1–10 km and up) and limited in temporal coverage (typically no more recent than 2006; S1 Table) [17,44–46]. As we are interested in areas undergoing rapid changes, we here opted to forgo the advantages of a proper land use classification, in favour of GlobeLand30-2010’s higher spatial resolution and more recent temporal coverage. In doing so, we test the hypothesis that deforestation for pasture can be captured by the Grassland class of GlobaLand30-2010 in the post-loss land cover analysis.

The official year for the GlobeLand30-2010 product is 2010, but in practice, the remote sensing data underlying the final product are from a range of years (e.g. due to cloud cover) [21]. For Latin America, it varies between 2006 and 2012 (Fig A in S1 Appendix), so in our analysis, we also include the year of the GlobeLand30-2010 data for each pixel, using this to ascertain the relative timing between forest loss and the land cover assessment.

To evaluate the approach taken here to assess post-loss land cover, we use two additional datasets over the Brazilian Legal Amazon: Projeto de Monitoramento do Desmatamento na Amazônia Legal por Satélite (PRODES), which provides spatially-explicit annual deforestation estimates for 2001–2014, and TerraClass for 2010, which maps land cover and use in the Brazilian Legal Amazon [47,48]. Also these two datasets are based primarily on Landsat data, and are provided by the Brazilian National Institute for Space Research (INPE) and the Brazilian Agricultural Research Corporation (Embrapa). PRODES monitoring is since 2008 used to enforce the Plan for Preventing and Controlling Deforestation in the Amazon, and a notable difference between PRODES and GFC is that PRODES exclusively considers loss of primary forest. Unlike GFC, PRODES also uses a minimum mapping unit of 6.25 ha (although patches of >1 ha are later included if they subsequently combine to exceed 6.25 ha) [47–50]. Unpublished accuracy assessments of PRODES (based on reference data from a few scenes of higher resolution RapidEye and SPOT data) indicate an overall accuracy exceeding 90% [51,52]. TerraClass uses detailed land use/cover classes, including Annual crops (Table 1) and several types of pasture. Its Non-forest class (from PRODES) is used for vegetation of different types, including savannah, shrub and forest vegetation of the Cerrado, Savana Gramíneo-Lenhosa, Lavrados and Campinarana [41,42,48]. TerraClass has an overall accuracy of 76%; however, if pasture classes are grouped (as we do here), it increases to 90% [47].

All datasets used here are freely available online, and data were downloaded from their respective online locations, reprojected, and slightly resampled or rasterised to match the GlobeLand30-2010 grid. The TerraClass and PRODES datasets were also slightly offset prior to rasterisation to align better with GlobeLand30-2010 and GFC. Once prepared, the datasets were combined pixel-by-pixel to create a single map where each pixel contained information from all input datasets. This was done in two versions: a “two-dataset” map for the whole of Latin America and the Caribbean, and a “four-dataset” map over the BLA (limited to the common extent of all the datasets). The “two-dataset” map pixels thus contain information on the year of GFC forest loss, and GlobeLand30-2010 class and year, while the “four-dataset” BLA map pixels additionally include the year of PRODES deforestation and the TerraClass 2010 class. The results were then compiled and compared at different at levels of geographical detail, including countries, biomes (for the BLA) as well as a hexagonal grid (for visualisation).

### 2.4 Quantifying and assessing post-loss land cover

Post-loss land cover was allocated to pixels with 2001–2011 GFC forest loss prior to the GlobeLand30-2010 year over that area. As the timing of the GlobeLand30-2010 data varies, it was not possible to directly assign post-loss land cover for all deforestation prior to 2010 in all places. Rather, depending on the year of the land cover data in each location, different forest loss years were considered; for example, where the land cover data is from 2006, the post-loss land cover could only be determined for forest loss 2001 to 2005, whereas where the GlobeLand30-2010 data is from 2012, post-loss land cover during the full time period could be considered. Where summarised by geographical units (such as country, biome or hexagon), any forest loss post-dating the GlobeLand30-2010 land cover data is assumed to follow the average share of allocated post-loss land cover within that geographical unit.

To determine what number of years between forest loss and the land cover assessment would be suitable for assigning post-loss land cover, we varied this lag to test how it impacted the results. For most countries, the post-loss land cover is not very sensitive to the lag time variations between 1 and 10 years (S2 Appendix). This is consistent with findings of Morton et al. [53], who find that conversion to cropland and pasture occurred rapidly after deforestation in the southern Brazilian Amazon. We thus assign post-loss land cover to all pixels where forest loss occurred up until the year prior to the land cover assessment (e.g. where GlobeLand30-2010 is for year 2011, we consider forest loss 2001–2010), as this gives the largest number of pixels to base results on. As such, the proportions of post-loss land cover are, especially in some areas, more heavily based on earlier parts of the time period. In total, post-loss land cover could be directly allocated to 71% of the 2001–2011 forest loss in Latin America (S2 Table).

To evaluate the approach taken here to quantify the relative contribution of pasture and cropland expansion into forests, we compare the results at country level to previous literature, and a more detailed comparison was made for the BLA using the “four-dataset” map. For the BLA, post-loss land cover was determined for all four combinations of datasets (GFC or PRODES, followed by GlobeLand30-2010 or TerraClass), to assess the sensitivity to choice of dataset. We further examine temporal and spatial correspondence between the forest loss/deforestation datasets, and differences in what land cover types they capture forest loss in.

In addition, to gain a regionally-specific assessment of GlobeLand30-2010’s land cover classification, we compared GlobeLand30-2010 with the higher accuracy TerraClass 2010 land cover/use datasets. While the differing classes and timing between GlobeLand30-2010 and TerraClass do not allow for a straightforward comparison, we examined the agreement between their classes using cross-tabulations. We did this both for (1) the datasets on a whole, and (2) for only those pixels that underwent forest loss. To reduce differences arising from actual land cover changes occurring between the timing of the two datasets, we in (1) excluded pixels where either GFC and/or PRODES show forest loss between the timing of GlobeLand30-2010 and TerraClass. In (2), we only include pixels with forest loss before 2010 and prior to the timing of the GlobeLand30-2010 data. These comparisons between GlobeLand30-2010 and TerraClass also let us test how cropland and pasture are represented in the land cover classes of GlobeLand30-2010 (primarily Cultivated land and Grassland).

Finally, to examine the potential effect of higher error rates at class boundaries, we assessed the relationship between likely errors in either of the global datasets and the proportion of class boundaries over a hexagonal grid.

## 3 Results and discussion

### 3.1 Post-loss land cover across Latin America

**Fig 1:**
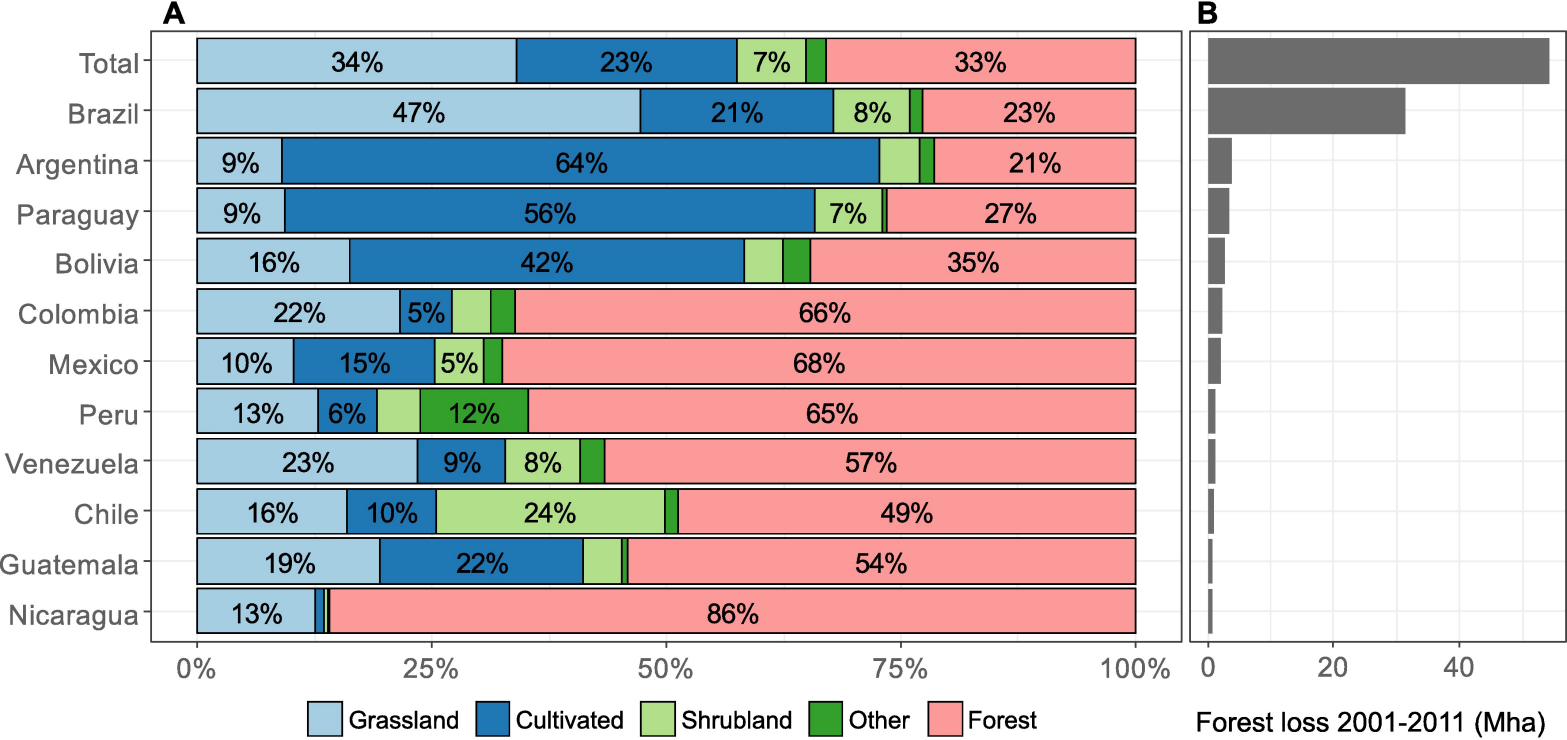
Forest loss 2001–2011 and post-loss land cover per country. (A) Proportion of GlobeLand-30-2010 land cover types following GFC forest loss. The proportions are based on forest loss for part of the period only, as post-loss land cover can only be assessed for areas with forest loss prior to the date of the land cover data. (B) Tree cover loss 2001–2011 per country, detected by GFC.

For the whole of Latin America, Grassland and Cultivated land accounted for roughly a third and a quarter, respectively, of land cover following forest loss (Fig 1). In Brazil, where the majority of the forest loss occurred, just under half of the post-loss land cover was classified as Grassland, while Cultivated land was found following just over a fifth of the forest loss (S3 Table). Cultivated land, according to our results, was found to be the dominant post-loss land cover in Argentina, Paraguay, and Bolivia (which also have had large amounts of forest loss). Somewhat surprisingly, as can be seen in Fig 1, it is also clear that GFC forest loss is frequently followed by a notable amount of land cover still classified as forest by GlobeLand30-2010 (though less so in the four countries with the most forest loss). Potential explanations for this are examined further in Section 3.4.

**Fig 2:**
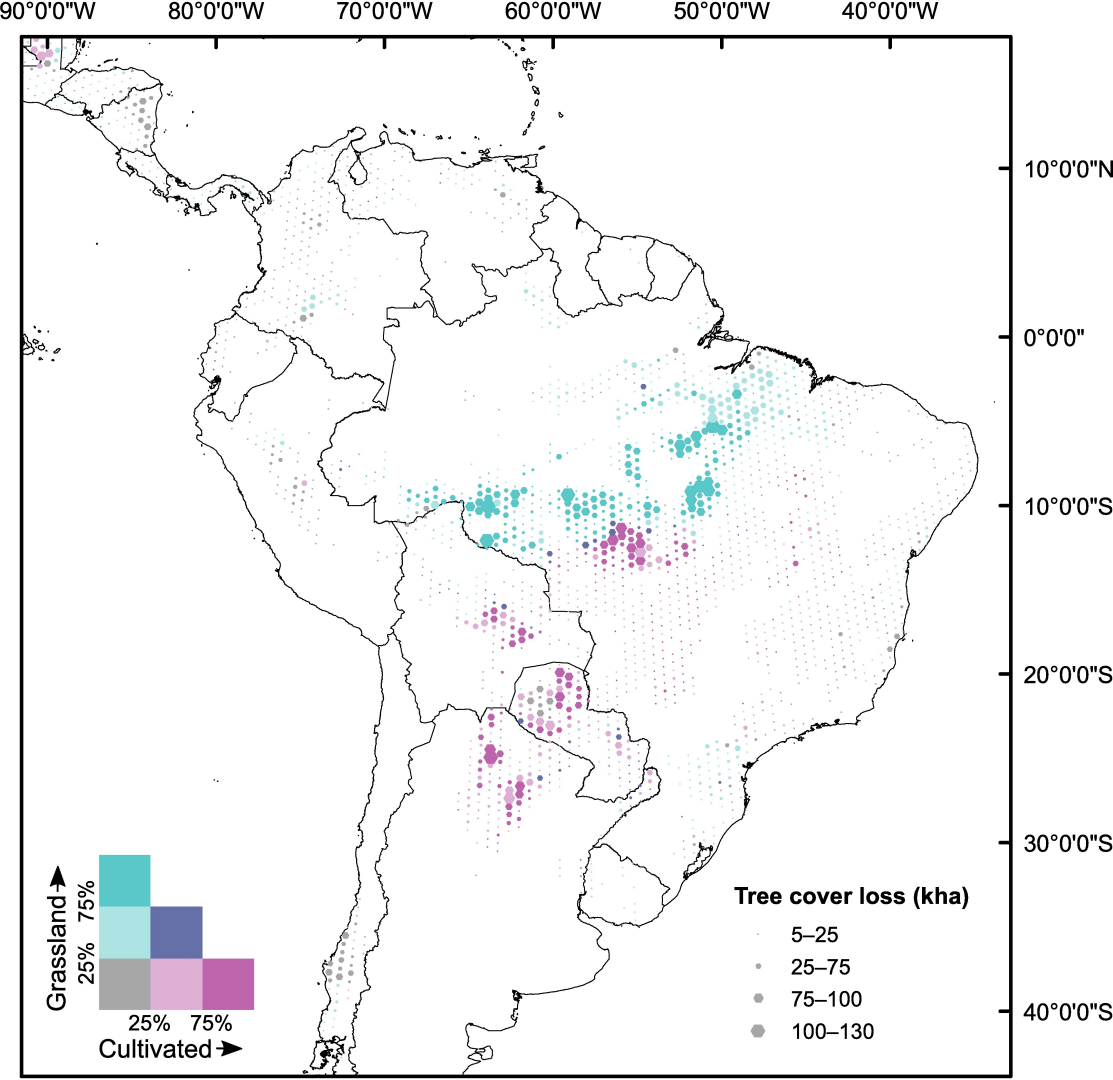
Accumulated GFC tree cover loss 2001–2011 and post-loss GlobeLand30-2010 land cover. As in Fig 1, the proportions of Grassland and Cultivated land are based on part of the time period only.

In Brazil, forest loss is concentrated along the “Arc of Deforestation” (Fig 2), with forest loss in the northern parts of this predominantly followed by Grassland, and by Cultivated land in the southern parts (in the Mato Grosso seasonal forests and the Cerrado). The spatially-explicit results show a clear boundary between where forest loss is followed by Cultivated land, and where it is followed by Grassland (rather than by a mixture of the two).

Other areas with large amounts of forest loss 2001–2011 include the Dry Chaco in northern Argentina and northwest Paraguay, and the Bolivian lowlands (in particular in the Chiquitano Dry Forest), where Cultivated land is found to be the dominant post-loss land cover (Fig 2).

**Fig 3:**
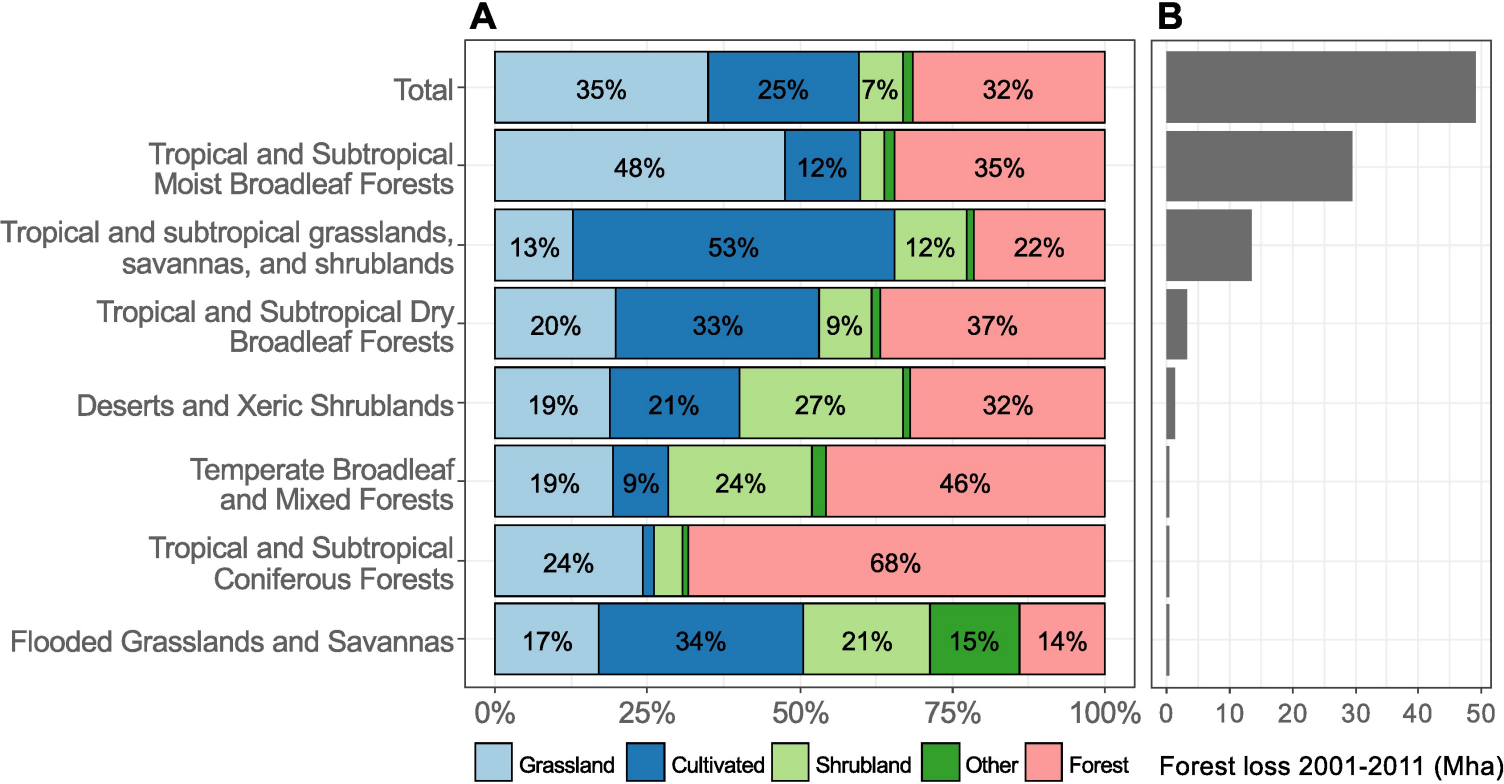
Forest loss 2001–2011 and post-loss land cover per biome. (A) Proportion of GlobeLand-30 land-2010 cover types following GFC forest loss. (B) Tree cover loss 2001–2011 per biome, detected by GFC (only the biomes with the most forest loss are shown). Biome boundaries from Terrestrial Ecoregions of the World [54].

At the biome level (Fig 3), most of the forest loss in Latin America occurred in Tropical and Subtropical Moist Broadleaf forests (constituted primarily of the Amazon and Atlantic forests, and the Petén-Veracruz moist forests), as well as in tropical and subtropical grasslands, savannas and shrublands (constituted primarily of the Cerrado, and the Dry and Humid Chaco). Grassland is the most common post-loss land cover (48%) in moist broadleaf forests, whereas in grasslands, savannas and shrublands, Cultivated land accounts for most (53%) of the land cover following forest loss. A further detailed breakdown per ecoregion is provided in S3 Appendix.

### 3.2 Comparison with previous studies

The relative share of pasture and cropland following forest loss found by combining GFC and GlobeLand30-2010 differs partly from previous studies at the continental and national/regional level. While Grassland–which we here interpret as pasture–is found to be the most common post-loss land cover at 34% (on average for Latin America), previous studies (using more specific land use classes) ascribe a larger role to Pasture. For instance, De Sy et al. [23] attribute 69% of 2000–2005 deforestation in South America to Pasture, and only 19% to commercial and smallholder cropland (quite similar to the 24% we find for Cultivated land).

Argentina is the country where our approach attributes the largest share (64%, 2.5 Mha) of forest loss to Cultivated land (concentrated in the Gran Chaco) (Fig 2). Other studies also find that Cropland, mainly for soybean, was one of the main land uses expanding into forests in Argentina (e.g. [5,55,56]). Cropland expanded until 2007 (although primarily into pastures), and contributed to deforestation, especially in the Chaco [24]. De Sy et al. [23] attribute 43–45% of deforestation to expansion of Pasture and Commercial crop respectively, while Henders et al. [5] attribute 70% of deforestation to soybean expansion (based on agricultural statistics) and 20% to cattle. Based on this it is reasonable that our approach finds Cultivated land to be the main land cover following forest loss, however, the post-loss Grassland share found in our study (9%) is somewhat lower than expected. In Paraguay, most of the forest loss occurred in the Chaco, with a smaller share in the Atlantic Forest (Fig 2). The post-loss land cover found differs significantly from other studies. Our approach indicates that Cultivated land is dominant (56%, 1.2 Mha) and Grassland is ascribed only a smaller share (9%, 0.3 Mha). Most other studies [23,31,55,57] based on remote sensing and/or agricultural census data, find that pasture expansion is the dominant proximate cause of deforestation in Paraguay (e.g. >70% in De Sy et al. [23]), especially in the Chaco biome. In the Atlantic Forest, cropland expansion (in particular for soy), did contribute to deforestation [5,24,58]. However, in the Paraguayan Chaco, the area of land cultivated with annual and perennial crops (based on agricultural census data) decreased between 1991/1992 and 2008/2009 [57] and while there was a slight increase (0.06 Mha) in cropland area at the expense of forest between 2001 and 2012 [31], this area is minimal compared to the total deforestation.

For Bolivia, GlobeLand30-2010 also attributes a lesser share of GFC forest loss to Grassland (16%, 0.4 Mha) than what previous literature on causes of deforestation in Bolivia ascribe to pasture. A spatially-explicit study by Müller et al. [59] find that cattle ranching on cultivated pastures account for 52% (0.94 Mha) of 2000–2010 deforestation (rising from 44% during 2000–2005 to 60% during 2005–2010). De Sy et al. [23] similarly attribute 39% of 1990–2005 deforestation to pasture. The share of Cultivated land (42%, 1.2 Mha) found with our approach, however, is more similar. Müller et al. [59] attribute 48% (0.87 Mha) to agriculture (of which approximately 62% is mechanized agriculture and 38% small-scale agriculture), although with a lesser role (40%) in the second half of the decade, down from 56% in 2000–2005.

In summary, the approach taken here generally seems to ascribe a larger share of land cover/use following forest loss to cultivated land than other studies ascribe to cropland. It also generally underestimates the share of grassland / pasture. A main explanation for the results found with our approach, is that GlobeLand30-2010’s defines its Cultivated land class to include certain types of pasture [21]. In Bolivia, cultivated pastures are dominant [59,60], and some of these can therefore be expected to be included under GlobeLand30-2010’s Cultivated land rather than Grassland. Likewise, in the Paraguayan Chaco, it is also likely that most of what GlobeLand30-2010 has classified as Cultivated land is mainly pasture (rather than cropland). Previous studies have highlighted the difficulty of achieving a reliable distinction between cropland and pasture in remote sensing analyses in general, especially in savanna regions [61,62]. For example, in the Paraguayan Chaco, crops and grasses can be spectrally similar, and therefore challenging to distinguish in remote sensing data [31,57]. There have been some recent advances in dealing with spectral similarities between classes (combined with spectral heterogeneity within classes), e.g. using time-series data to help distinguish pastures from croplands based on differences in phenological variations of the vegetation over the year [61]. However, there is still a lack of consistent, spatially-explicit data on cropland and pasture, which is further complicated by ambiguities arising from semantic differences between the classes [17,61,63,64].

### 3.3 Comparison with datasets over the Brazilian Legal Amazon

For the Brazilian Legal Amazon, INPE’s TerraClass project monitors land cover and land use following deforestation detected by PRODES [42]. Both PRODES and TerraClass have higher reported accuracies than GFC and GlobeLand30-2010 (~90% compared to ~80%), and PRODES is also used to enforce the Plan for Preventing and Controlling Deforestation in the Amazon (as noted in Section 2.3). It is therefore interesting to analyse how the results presented here using GFC and GlobeLand30-2010 agree with these datasets, both in terms of rates and location of forest loss, as well as the post-forest loss land cover.

Previous studies using PRODES and TerraClass have shown that pasture is the main land use (60–80%) following deforestation, while cropland covers very little (2–5%) of deforested areas [42,65,66]. Our analysis, using these datasets and employing the same method for analysing post-loss land use as for the global datasets, give similar results (Table 2). (These results are also similar to those found by [66], who use a sample-based approach.) More importantly, for the Brazilian Legal Amazon as a whole, the approach of combining GlobeLand30-2010 and GFC for assessing the share of land cover following forest loss gives results that overall are very similar to the TerraClass project, especially for the Grassland / Pasture class. For the other land cover classes the results differ somewhat; for example Cultivated land is generally ascribed a larger share of the forest loss than does the Annual crops class in TerraClass, while the Forest class comes out as more significant in the global dataset analysis than in that using PRODES and TerraClass.

**Table 2:** Comparison of post-loss land cover/use for the Brazilian Amazon using different combinations of datasets.

*Forest loss dataset*:	GFC	PRODES	GFC	PRODES
*Land cover /use dataset*:	GlobeLand30	GlobeLand30	TerraClass	TerraClass
*Post-loss land classification*:				
Grassland (GL) / Pasture (TC)	60%	55%	56%	59%
Cultivated land (GL) / Annual crops (TC)	15%	11%	7%	7%
Forest (GL & TC)	18%	27%	11%	5%
Other (GL & TC)	7%	7%	7%	8%
Non-forest / Secondary vegetation (TC)			20%	21%

Results from combining different global and regional forest loss datasets—Global Forest Change (GFC) and PRODES (2001–2009)—and land cover / land use datasets—GlobeLand30-2010 (GL) and TerraClass (TC).

Part of the differences between our results and the TerraClass project are due to semantic differences between both the forest loss and the land cover/use datasets. For example, GFC (used in our study) and PRODES (which TerraClass uses) use different definitions of forest and forest loss (Table 1): while PRODES only considers loss of primary forest [41,42], GFC also counts clearing of secondary vegetation and planted forest [19,39]. To examine how these semantic differences between GFC and PRODES affect post-loss results, we here compare forest loss according to both datasets within their joint extent, and assess temporal and spatial differences between them.

**Fig 4:**
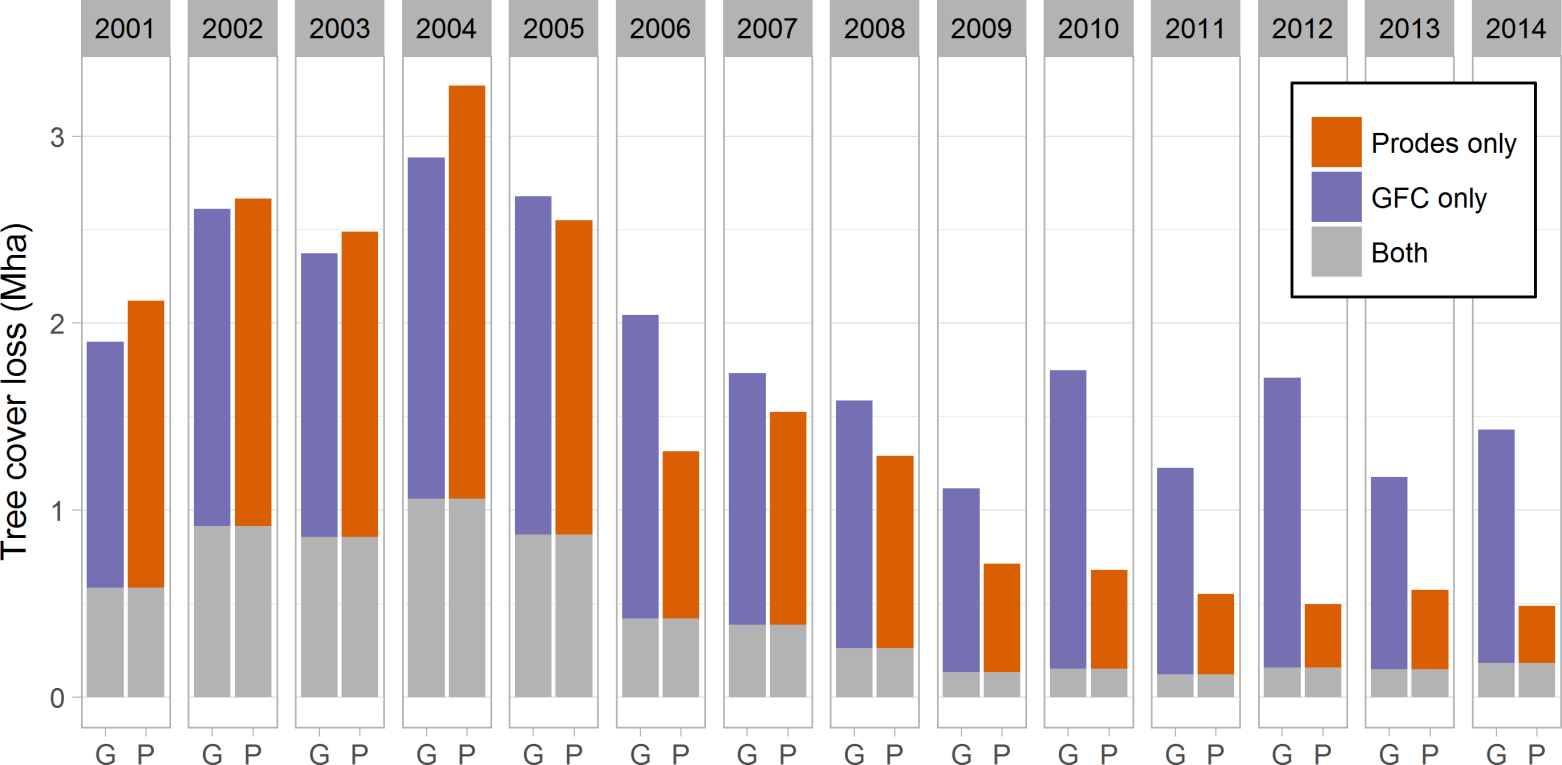
Annual GFC (G) and PRODES (P) forest loss in the Brazilian Legal Amazon 2001–2014. Grey shows where both datasets report loss at some point during the time period.

Forest loss peaks in both datasets in 2004, and until 2008 both datasets show reductions and similar amounts of forest loss (Fig 4). In 2009 the dataset series start to diverge and GFC estimates more than double the forest loss of PRODES during 2010–2014. However, even prior to the divergence between the two datasets in terms of total estimated deforestation, the exact location (at pixel level) differs for much of the forest loss: looking at the full time period, only 54% of the (26.2 Mha) GFC forest loss is also found in PRODES, and 68% of the (20.7 Mha) PRODES deforestation is found in GFC. Thus, spatial differences account for a majority of the discrepancies between the differences. This has previously been shown also by Richards et al. [49] and Fanin et al. [67].

S1 Fig reveals that the two datasets agree best on the location of forest loss in the southern parts of the Amazon biome. In the easternmost parts of the Amazon, both datasets show forest loss but exhibit large differences in location. As expected, agreement is especially poor in the Cerrado biome (S2 Fig), as PRODES by definition does not consider forest loss for much of the vegetation in this biome.

The different forest and forest loss definitions used by PRODES and GFC are also visible in the data when looking at the land cover prior to the forest loss: in TerraClass forest, they both capture similar amounts of losses (1.2 Mha in GFC and 1.0 Mha in PRODES), but GFC also captures additional losses in secondary vegetation/non-forest (0.9 Mha) and pasture (0.6 Mha) (Table D in S1 Appendix). Thus, part of the larger losses seen in the GFC dataset, could also be the reclamation or intensification and clearing of previously abandoned land. This could be a consequence of increased regulation and enforcement shifting deforestation away from primary forest (and/or the areas monitored by PRODES) [49,68]. There is also some disagreement between the datasets on the timing of forest loss occurrences. However, much of the forest loss that is captured by both datasets is reported within a few years of each other (S4 Table and Fig E in S1 Appendix).

The quantification of pasture and cropland expansion into forests is clearly also highly dependent on the chosen land cover classification, in this case that of GlobeLand30-2010. To evaluate how GlobeLand30-2010’s Grassland and Cultivated land correspond to Pasture and Cropland, we examine the agreement between GlobeLand30-2010 and the higher accuracy TerraClass 2010 on a pixel-by-pixel basis, for all common areas, as well as only for pixels classified as forest loss by GFC.

Table 3 shows a cross-tabulation between TerraClass 2010 and GlobeLand30-2010 for all areas within their common extent. The best correspondence is found for the forest classes (> 90%), indicating that GlobeLand30-2010 and the more regional TerraClass agree well on the location of forests in the Brazilian Legal Amazon. While GlobeLand30-2010’s Grassland does capture most (74%) of the TerraClass Pasture, and Cultivated land most (83%) of the Annual crops (i.e. the Producer’s agreement is quite good), these GlobeLand30-2010 classes have high levels of commission of other classes. Half of what GlobeLand30-2010 classifies as Grassland is Pasture, but it also includes large amounts (32%) of Non-forest (S5 Table). GlobeLand30-2010’s Cultivated land predominantly includes Non-forest (60%) and Pasture (20%), and only 16% consists of Annual crops as classified by TerraClass.

**Table 3:** TerraClass 2010 and GlobeLand30-2010 land cover/use (Mha) for all pixels.

*GlobeLand30*:	Grassland	Cultivated	Forest	Other	Total	Agreement (Producer's)
*TerraClass 2010*:						
Pasture	27.5*	3.8	3.7	2.4	37.4	74%
Annual crops	0.4	2.8*	0.1	0.1	3.4	83%
Forest	2.3	0.1	288.6*	22.0	313.0	92%
Other	2.4	0.1	2.8	9.8	15.1	
Non-forest / Secondary vegetation	19.7	11.2	22.2	36.1	89.2	
Total	52.3	18.0	317.4	70.4		
**Agreement (User's)**	53%	16%	91%			

Based on all pixels within the common extent, except those where GFC and/or PRODES show forest loss between the timing of TerraClass and GlobeLand30-2010.

Agreement between the datasets shown for the classes indicated with an asterisk (*).

Note that while TerraClass has a higher accuracy than GlobeLand30-2010, the disagreement between the two datasets can be due to errors in either of them.

This indicates that a straightforward assumption that GlobeLand30-2010’s Cultivated land equals Cropland cannot be made, as it also includes large amounts of natural vegetation and pastures. Note that this does not necessarily reflect an error in GlobeLand30-2010, as the Cultivated land class, as discussed in 3.2, is constructed to include some pastures and abandoned arable land, but rather indicates that a simple translation into cropland is not advisable. Nor can Grassland generally be assumed to be pasture.

However, if we look specifically at pixels that previously underwent forest loss (Table 4) the agreement between Grassland/Pasture and Cultivated land/Annual crops classes improves. Three-quarters of the Grassland following forest loss is indeed Pasture according to TerraClass, with the remainder mainly consisting of secondary vegetation and non-forest (12%). This indicates that our hypothesis that GlobeLand30-2010’s Grassland can be equated to Pasture is reasonable where it follows forest loss.

**Table 4:** TerraClass 2010 and GlobeLand30-2010 land cover/use classes (Mha) following GFC forest loss.

*GlobeLand30*:	Grassland	Cultivated	Forest	Other	Total	Agreement (Producer's)
*TerraClass 2010*:						
Pasture	7.4*	0.6	0.9	0.3	9.3	80%
Annual crops	0.1	1.1*	0.0	0.0	1.2	87%
Forest	0.6	0.1	0.7*	0.2	1.6	46%
Other	0.6	0.0	0.3	0.1	1.1	
Non-forest / Secondary vegetation	1.2	0.8	1.0	0.5	3.4	
Total	9.9	2.5	3.0	1.1		
**Agreement (User’s)**	75%	43%	24%			

Includes GFC forest loss prior to 2010 (and to the year of GlobeLand30-2010 land cover data).

Agreement between the datasets shown for the classes indicated with an asterisk (*).

Cultivated land (following forest loss), although having a low omission (13%) of Annual crops, consists of less than half (43%) of Annual crops, and includes approximately a third Non-forest and a quarter Pasture. This indicates that the area of Annual crops/cropland will likely be overestimated when using GlobeLand30-2010 in this area (and, as noted previously for other parts of Latin America, expected to include some pasture).

For the pixels following forest loss, the Forest class exhibits the poorest agreement (24–46%). The poor correspondence of the forest classes is likely related to an overrepresentation of errors, as we here look specifically at areas that according to GFC underwent forest loss.

Table 2 illustrates how the abovementioned differences in the forest loss and land cover/use datasets affect the overall attribution of post-loss land cover, using all four possible combinations of forest loss and land cover datasets for their common extent in the BLA. As already noted, the largest differences are found between the post-loss forest class (18–27% in post-loss GlobeLand30-2010 compared to 5–11% in post-loss TerraClass), and between post-loss Cultivated land/Annual crops (11–15% compared to 7%). The larger amount of post-loss Forest in GlobeLand30-2010 is in part due to what in TerraClass is classified as Secondary vegetation and Pasture (S5 Table). The larger share of Cultivated land compared to Annual crops reflects that GlobeLand30-2010’s Cultivated land, as noted above, is more inclusive than Annual crops and likely provides an overestimate if Cultivated land is “translated” into cropland. However, overall, combining GFC and GlobeLand30-2010 gives similar results to PRODES and TerraClass (e.g. [42]), indicating that this comparatively simpler approach still provides quite decent results for the BLA.

### 3.4 Post-loss land cover classified as forest

As noted in the main results above, what seems to be a key limiting issue with the approach of combining GFC forest loss with subsequent GlobeLand30-2010 land cover is that a large share of the GFC forest loss is still classified as forest by GlobeLand30-2010 (Fig 1). When averaged for the whole of Latin America, approximately a third of post-loss land cover is classified as forest, still allowing room for quantifying the relative share of post-loss cropland and pasture for roughly two thirds of the (detected) forest loss. However for many countries, except for those with most forest loss, forest comes up as the dominant post-loss land cover (such as Colombia and Peru), and for Nicaragua the share is over 85%. This makes it challenging to ascribe the forest loss to e.g. pasture or cropland expansion based on the post-loss land cover. It also indicates some form of disagreement between the two datasets, as GFC forest loss should reflect the “*removal or mortality of all tree cover”* [39].

Alas, this high proportion of forest following forest loss does not, in most cases, signify that regrowth has occurred. If this were the main reason for the post-loss forest, one would expect the share of post-loss forest to increase with an increased lag between the forest loss and the land-cover assessment. However, for most countries, increasing this interval does not show a concomitant increase in the post-loss forest proportion (S2 Appendix). There are a couple of exceptions, with Chile being the clearest example. Here the data show a clear increase in the proportion of land classified as forest as the time from forest loss increases. This is corroborated by previous literature, reporting that during this time period, Chile greatly increased its area of forest plantations for timber, partly at the expense of secondary native forest [69–71]. A similar pattern is found for Uruguay, which also has large areas of (primarily eucalyptus and pine) plantations [72]. As the GFC dataset does include harvesting of plantations as forest loss (as noted by several authors, e.g. [19,39,73,74]), part of the post-loss forest seen in Chile and Uruguay is likely a result of this, rather than because of a land-use change. These countries are however exceptions and, in general, the high proportion of post-loss forest does not appear to be due to regrowth.

Part of the explanation lies in errors in the input datasets: although their overall accuracies exceed 80%, the total accuracy of the datasets combined will be lower than their individual accuracies. Disregarding any inaccurate alignment, and assuming that the errors are randomly distributed in space and in between classes (although this is likely not the case), their combined accuracy can be approximated by the product of their respective overall accuracies [75,76]. That is, given that the commission error for GFC loss has been estimated to 10–21% for the Latin America and the tropics/sub-tropics [19,77], and GlobeLand30-2010’s commission error is approximately 15–30% depending on the class [21], the probability that both classifications are correct in a given pixel with detected GFC forest loss would be approximated somewhere in the range 55–77%. This can explain much of the post-loss forest.

Furthermore, the classification accuracy is likely lower at class boundaries, for example due to mixed pixels [27,77,78]. As we specifically look at areas where land-cover change occurred recently (from forest to something else), many of these areas will be at the boundary between classes, and thus the accuracy is likely lower than might expected of the combination of the input datasets (if errors are assumed to be randomly distributed, as above).

To assess the relationship between post-loss forest and class boundaries, the proportion of boundary pixels in each hexagon was compared to the proportion of pixels with forest as post-loss land cover (i.e. where at least one of the datasets is likely incorrect). We find that high rates of “post-loss forest” are indeed more likely to occur where there is a greater amount of fragmented forest loss and/or land cover class boundaries (Fig H in S1 Appendix). This indicates that the approach (of combing global datasets) can be expected to be less reliable for areas with small-scale forest loss and in areas with heterogeneous land cover.

## 4 Conclusions

In this study, we have attempted to quantify where cropland and pasture expansion have replaced forests in Latin America and evaluated to what extent the recent global datasets Global Forest Change and GlobeLand30-2010 can be used for this purpose. We found that this approach does capture some of the major patterns of where pasture and cropland have expanded into forests, with GlobeLand30-2010’s Grassland class (which we interpret as pasture) being the most common land cover replacing forests across Latin America in the 2001–2011 period. The comparison with other studies shows that the estimates of land use following forest loss at national or regional level are for some areas similar to those produced with more advanced approaches (e.g., PRODES and TerraClass for the Brazilian Legal Amazon).

More importantly, the spatially-explicit results show that the land-cover changes following deforestation may vary substantially within and between countries, highlighting the need to account for spatial variations in land use dynamics when designing forest conservation policies, rather than assuming a single dynamic within a whole country.

The comparison between the global and the Brazilian datasets also point to the need for careful consideration in choosing the data to base, enforce or evaluate forest conservation policy on. Even though different datasets on the surface may seem to present similar information, there are often non-trivial differences between the classes (e.g. in the definition of forests, deforestation or cropland) which can lead to quite different results. For example, in the Brazilian case, PRODES excludes forest loss outside of primary forests and in larger patches, thus missing, for example, clearing of secondary vegetation and smaller patches adjacent to existing fields (potentially a response of land owners to increased enforcement tied to PRODES [49,79]) that is picked up by GFC. The point here is not whether one of the datasets is better than the other, but rather that the choice of dataset should be made with consideration for the intended purpose and with potential unintended perverse incentives in mind.

Our analysis also reveals some major limitations in using currently available global land cover datasets for large-scale assessments of land-cover transitions following forest loss. First, in general, the comparison with previous literature suggests that the approach taken here leads to an underestimation of the role of pasture in replacing forests in Latin America. The reason for this is that pasture and cropland are not defined to be entirely separated between GlobeLand30-2010’s Grassland and Cultivated land classes. The comparison with TerraClass, as well as the results for Paraguay, indicates that GlobeLand30-2010’s Cultivated land class in some areas contains not only cropland, but also large amounts of cultivated pastures. As a consequence, a straightforward “one-to-one legend matching” from Grassland to pasture (following forest loss), generally leads to an underestimation of the role of pastures in replacing forests. A further important implication is that GlobeLand30-2010’s Cultivated land class ought not to be directly interpreted as cropland (as is done by e.g. Lambert et al. [43], or by Jokar Arsanjani et al. [25] that suggest this dataset can be used to help improve global cropland maps) without careful attention to whether its definition is suitable for the intended application.

Second, the large share of forest loss identified by the Global Forest Change dataset still classified as forest by GlobeLand30-2010 points to difficulties of combining global datasets in general, and also suggests specific limitations with the datasets used here. Not only is the accuracy of combined datasets lower than the accuracy of the individual datasets, but also–particularly for our analysis across Latin America–the accuracy of the land cover classifications seems to be particularly poor in areas exhibiting small-scale and/or fragmented deforestation, and in areas with heterogeneous land cover (e.g. Nicaragua). Low accuracy of land cover classification in heterogeneous areas has also been noted for other land cover datasets [80]. Here our analysis suggests that the accuracy of the combined datasets is in most cases too poor for deriving an accurate quantification of the relative contribution of pasture and cropland expansion at the expense of forests.

Taken together, these points suggest that in spite of the recent advances in remote sensing and land cover mapping, identification of land-cover transitions using remote sensing data on continental to global scale still remains a significant challenge. There are however recent efforts at repeated mapping of land cover and land use at regional and national scales, such as MapBiomas [81], following earlier targeted mapping exercises with limited temporal and geographical scope (e.g. [23,31,32,82,83]). In lieu of a low-cost, repeatable approach for global assessments of the proximate causes of deforestation, forest conservation policy and recent initiatives for increased agricultural supply-chain sustainability will have to rely either on such efforts, or on broader but coarser analyses relying on a combination of e.g. deforestation data and agricultural statistics (e.g. [5,36,84–86]).

## Supporting information

S1 Table Overview of datasets considered for this study. (XLSX)

S2 Table GFC forest loss per year that could be attributed a post-loss land cover class. (XLSX)

S3 Table Post-loss GlobeLand30 land cover, and attributed GFC forest loss/country. Total GFC forest loss 2001–2011 per country (for the 11 countries with most forest loss) and share of this for which a post-loss land cover could be assessed. Area and share per each post-loss land cover class per country.
(XLSX)

S4 Table Cross-tabulation of GFC forest loss and PRODES deforestation (in Mha). Within the common extent of the datasets.
(XLSX)

S5 Table Full cross-tabulations between GlobeLand30-2010 and TerraClass 2010. Cross-tabulations for all land cover/use classes (Mha). Both for all pixels within the common extent (except those where GFC and/or PRODES show forest loss between the timing of TerraClass and GlobeLand30) and exclusively for pixels following GFC forest loss.
(XLSX)

S1 Appendix Additional descriptions of data and methods. (PDF)

S2 Appendix Impact of lag time on post-loss land cover. (PDF)

S3 Appendix Post-loss land cover/ecoregion, maps of selected biomes and ecoregions. Maps over study area showing biomes and ecoregions from Terrestrial Ecoregions of the World.
(PDF)

S1 Fig Map comparing GFC forest loss and PRODES deforestation 2001–2014. PRODES deforestation and GFC tree cover loss detected during the years 2001–2014 in their common extent in the Brazilian Legal Amazon, as well as some close-ups. Grey shows where both datasets report loss at some point during the time period (differences in timing of the loss event are not distinguished for in this figure).
(TIF)

S2 Fig Annual tree cover loss per biome, based on GFC (G) and PRODES (P). For areas where both datasets were available (i.e. mainly the Amazon biome). Note change in scale of the y-axis.
(TIF)
