# Supplementary material for: Combining global land cover datasets to quantify agricultural expansion into forests in Latin America: Limitations and challenges
Source: PLoS One. 2017 Jul 13;12(7):e0181202. doi: 10.1371/journal.pone.0181202 (PMC5509295; doi:10.1371/journal.pone.0181202)
Supplement: S1 Appendix — (PDF) [file pone.0181202.s006.pdf]

S1 Appendix. Supporting information to "Can global datasets be used to support fine-scale assessments of deforestation causes in Latin America?"

Additional information on datasets and methods

GlobeLand30

The GlobeLand30-2010 dataset is a global, multi-class land cover dataset provided at 30-m resolution by the National Geomatics Center of China (NGCC) [1]. It is based primarily on Landsat TM/ETM+ (Thematic Mapper, and Enhanced Thematic Mapper) supplemented by HJ-1 data, that are classified into 10 land cover classes using a combination of pixel- and object-based classification, along with manual verification [1].

We use the 2010 version of GlobeLand30, for which the input data varies from 2006 to 2012 in Latin America (Fig A). There is also a GlobeLand30 product for the year 2000, which was not used in this study.

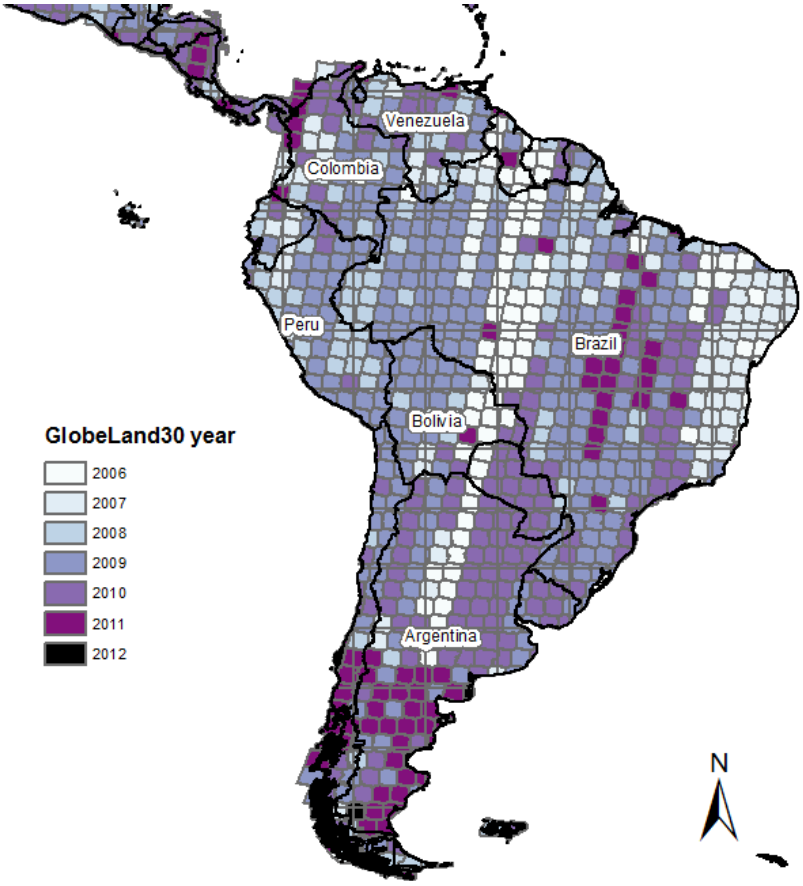

Fig A. Map showing the year of the Landsat data used for the GlobeLand30-2010 classification. Data set provided by the National Geomatics Center of China (DOI10.11769/GlobeLand30.2010.db) [1, 2].

Table A. Previous accuracy assessments of GlobeLand30.

Study	Overall accuracy	Coverage	Reference data	Class specific results
Chen et al. [1]	80%	Global	Based on 150 000+ test samples (GoogleEarth, TM images, etc.)	User's accuracies for Cultivated land (83%), Grassland (72%), Forest (84%). (Table 9 in [1].)
Brovelli et al. [3]	81 – 92% (5 CORINE classes; ~3–4 % better outside buffer.)	Italy	CORINE, Italian regional datasets (DUSAF)	
Jokar Arsanjani et al. [4]	74% (OSM) –92% (CORINE) agreement. Urban Atlas and ATKIS (best reference sources) > 85%.	Germany	CORINE, Urban Atlas, OpenStreetMap and ATKIS	Wetland poor
Jokar Arsanjani et al. [5]	78%	Iran	GoogleEarth sample points, OpenStreetMap, Flickr	Confusion matrix available
Lambert et al. [6]	88%	Sahelian and Sudanian agrosystems	Based on validation dataset from Zhao et al. [7], and 7 high resolution images.	
Yang et al. [8]	83%	China	GoogleEarth sample units	

GFC

The Global Forest Change (GFC) annual tree cover loss dataset is based on Landsat 7 ETM+ growing season data [9]. The commission error for GFC loss to 13% and 21% in the tropics and sub-tropics respectively, and omission errors of 17–21% in the tropics/subtropics [9]. In a more recent accuracy assessment (using multi-temporal Landsat and GoogleEarth imagery for 1200 30x30 m reference pixels) Tyukavina et al. [10] found even lower commission and omission errors for Latin America.

PRODES

Deforestation in the Brazilian Legal Amazon (BLA) has been monitored within the PRODES program by the Brazilian National Institute for Space Research (INPE) since 1988 [11]. We used this dataset on annual deforestation for 2001 to 2014. PRODES is primarily based on Landsat data July–September [12].

The PRODES data includes attributes on the year that deforestation was detected, as well as on when the area was last observed (e.g. if the area was covered by clouds in the preceding years). For example, d2001_4 is used for deforestation detected in 2001, in an area which had not been observed for four years prior. In our analysis we ascribe the deforestation to the earliest year of these (e.g. for d2001_4, we treat this as deforestation in 1997), to ensure that only pixels deforested within the analysis time period were included in the main analysis.

TerraClass

TerraClass is based primarily on the same Landsat imagery as PRODES, focused on the time period July–September [12].

TerraClass and GlobeLand aggregated classes look-up table

For some of the analyses, we use aggregated classes for GlobeLand30 and TerraClass. Tables A and B detail how the original classes were aggregated.

Table B. Look-up table of aggregated GlobeLand30 classes used in this study.

Aggregated GlobeLand30 classes	Original GlobeLand30 classes
Grassland	Grassland
Cultivated land	Cultivated land
Forest	Forest
Shrubland	Shrubland
Other	Artificial surfaces, Bareland, Snow and ice, Water bodies, Wetland, (Shrubland – for comparison to TerraClass only)

Table C. Look-up table of aggregated TerraClass-2010 classes used in this study.

Aggregated TerraClass classes	Original TerraClass classes
Pasture	PASTO_LIMPO, PASTO_COM_SOLO_EXPOSTO, PASTO_SUJO, REGENERACAO_COM_PASTO
Annual crops	AGRICULTURA_ANUAL
Forest	FLORESTA
Other	OUTROS, AREA_NAO_OBSERVADA, AREA_URBANA, DESFLORESTAMENTO_2010, HIDROGRAFIA, MINERACAO, MOSAICO_DE_OCUPACOES, REFLORESTAMENTO
Non-forest/ Secondary veg.	NAO_FLORESTA, VEGETACAO_SECUNDARIA

Administrative and biome boundaries

Country boundaries were retrieved from the GADM database of Global Administrative Areas via DIVA-GIS at http://www.diva-gis.org/Data/ (on 2015-08-21).

Biome boundaries for Brazil were obtained from the Brazilian Ministry of Environment (http://mapas.mma.gov.br/i3geo/datadownload.htm).

Data access and processing

Data processing and analysis was primarily carried out using Safe Software's FME Desktop (2015.1.1 Build 15515). Some reprojections were done in QGIS 2.16.0, as was the creation of an (equal size/area, approx. 3 200 km^2) hexagonal grid (using the mmqis plugin). Most figures were created in R with ggplot, and Excel, except the maps, which were created in Environmental Systems Research Institute's (Esri) ArcMap 10.2.

As we here use data that were are available online, data were downloaded from their respective online locations and reprojected as needed.

The Global Forest Change data version 1.2 were downloaded (on 18 December 2015) via http://earthenginepartners.appspot.com/science-2013-global-forest. The part of the dataset used here was the "year of gross forest cover loss event (lossyear)", provided in tiff format. The forest loss data were masked to where canopy cover in the year 2000 exceeded 30% (based on GFC treecover2000, downloaded on 20170112 using the R package gfcanalysis, 2015 version (v.1.2) of data).

The GlobeLand30-2010 data set is provided by National Geomatics Center of China (DOI10.11769/GlobeLand30.2010.db) and was ordered via http://www.globallandcover.com and subsequently downloaded (between November 2015 and March 2016). In addition to the geotiff files containing the land cover classification, the data were supplemented by a shapefile with information on the source Landsat tile used, which we use to gain information on the actual year the land cover classification was valid for.

The PRODES data were accessed via http://www.dpi.inpe.br/prodesdigital/prodes.php (in February 2016). The version used here was the 2014 version of the state mosaics in shape (vector) format. Only polygons marked (in "mainclass") as "desflorestamento" or "residuo" were used.

The full set of TerraClass data for 2010 were downloaded from http://www.inpe.br/cra/projetos_pesquisas/terraclass2010.php (except tiles 231/61 and 231/68, which were not available at the time of download on 2016-01-16).

In a visual examination of the datasets, PRODES and TerraClass align well with each other, as do GFC and GlobeLand30 (Fig B), but PRODES / TerraClass seemed somewhat offset relative to GFC / GlobeLand30. Therefore, PRODES and TerraClass data were offset by -0.001 degrees in longitude and 0.001 degrees in latitude. This improved the correspondence between PRODES and GFC, seen in the number of pixels classified as deforested by both datasets.

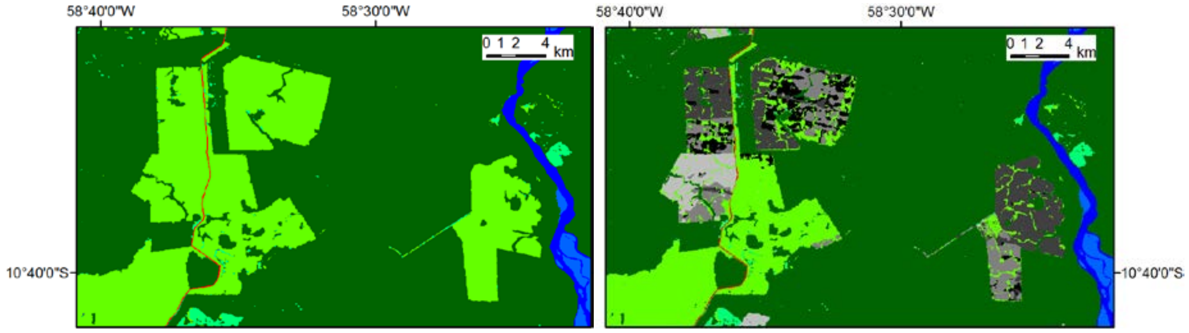

Fig B. Example of alignment between GlobeLand30 and GFC (in Mato Grosso, Brazil). Shown in simple overlay. Left: GlobeLand30 (based on 2006-data). Right: Same area with GFC forest loss 2001–2005 overlaid (in grey/black) on GlobeLand30 (Forest is shown in dark green, Grassland in bright green). GlobeLand30 dataset provided by the NGCC (DOI10.11769/GlobeLand30.2010.db) [1, 2], GFC forest loss data source: Hansen/UMD/Google/USGS/NASA.

To combine the datasets pixel-by-pixel, all datasets were aligned to the GlobeLand30 grid. To reduce the consequences of shifting when aligning raster pixels for the pixel-by-pixel comparisons, the GlobeLand30 data were rescaled to a third of their input pixel size (i.e. from c. 30 m to c. 10 m; this does of course not improve the actual resolution of data). PRODES and TerraClass were then rasterized to directly match this grid, and the GFC data slightly resampled (all resampling done using nearest neighbour) and/or offset, and the analysis performed at this

finer resolution. Pixel cell sizes were calculated in a Lambert Azimuthal Equal Area projection for each tile.

The map comparing overlap and differences between PRODES and GFC (S1 Fig) was created by reclassifying the pixels into three classes: PRODES only; GFC only; and "Both" for pixels labelled as deforested by both datasets at some point during the years 2001–2012. For this map, the comparison was made for the full time period, and any differences in timing between forest loss in the two datasets were not taken into account. For visualisation only, data were resampled to approx. 60-m resolution.

Shares of GFC forest loss that could be attributed a post-loss GlobeLand30 land cover

The amount and share of GFC forest loss that could be attributed a post-loss GlobeLand30 land cover per forest loss year are shown in Fig C and S2 Table. Amounts and shares are per country are shown in S3 Table, and per hexagon in Fig D.

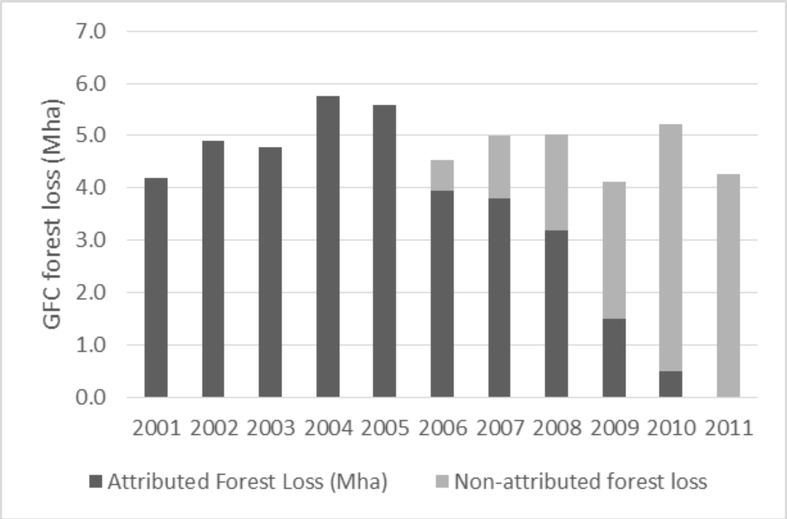

Fig C. GFC forest loss that could be attributed to a post-loss GlobeLand30 land cover class per year (as the GlobeLand30 land cover data post-dated the forest loss).

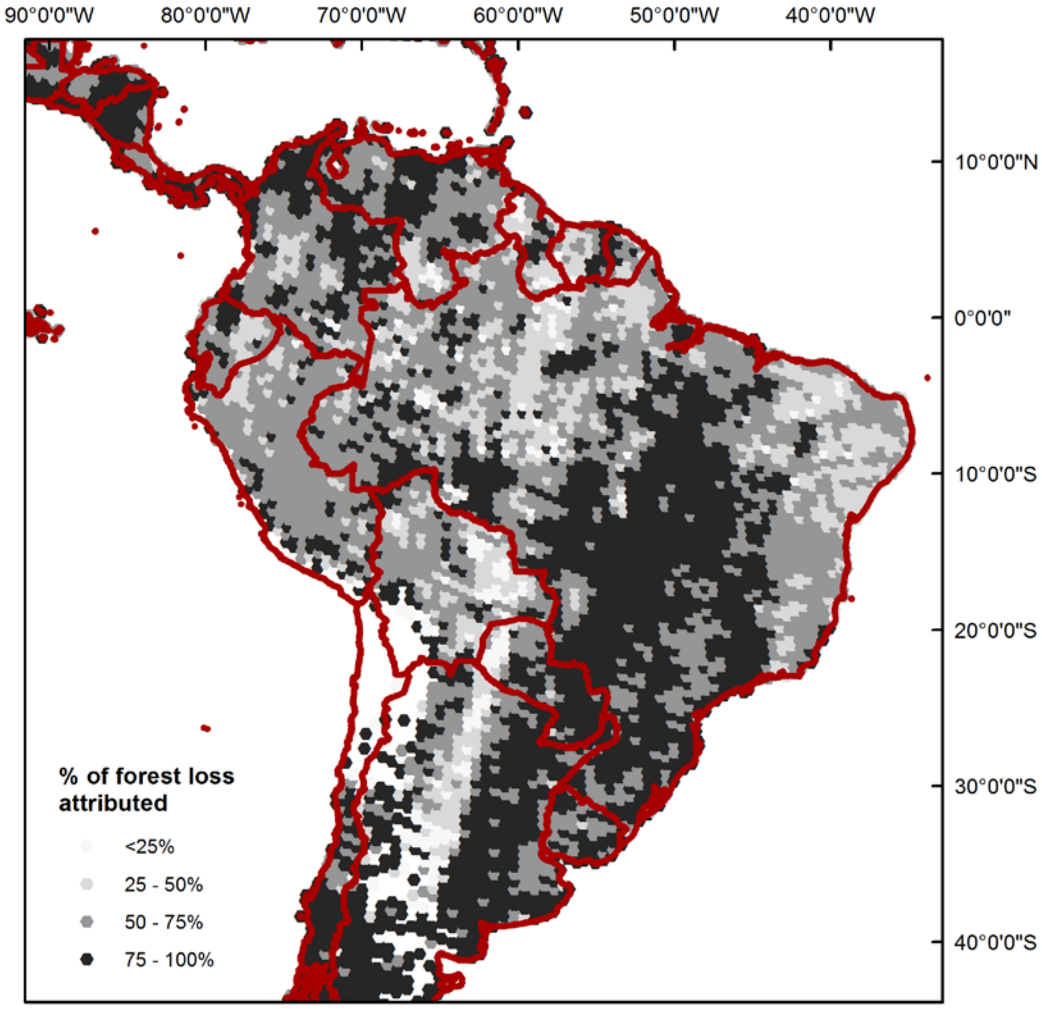

Fig D. Map of GFC forest loss percentage that could be attributed to a post-loss GlobeLand30 land cover class (as the GlobeLand30 land cover data post-dated the forest loss), per hexagon.

PRODES – GFC inter-comparison

Many pixels are marked as deforested by only one of GFC and PRODES, but for the pixels classified as forest loss by both datasets, the timing is in many cases within a few years of each other (Fig E and S4 Table). As Richards et al. [13] note, this can in part be due to the different time of year that the datasets are based on. A quarter of the additional GFC forest loss was found by Richards et al. [13] to be in areas smaller than PRODES minimum mapping unit (6.25 ha).

Similar comparisons between PRODES and GFC have been done by Fanin et al. [14] and Richards et al. [13]. Fanin et al. [14] perform their analysis at a coarser resolution (500 m). The analysis by Richards et al. [13] differ slightly from what is done here in that they use a 90-m raster version of PRODES (whereas we use the vector version) and also do not include deforestation for 2001.

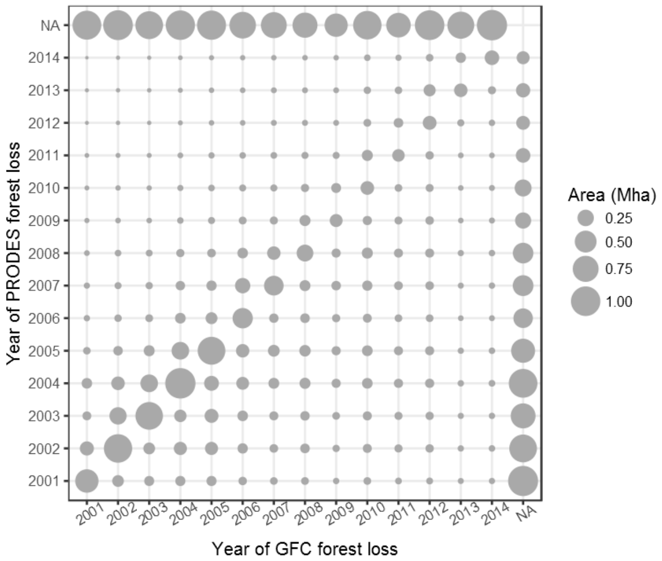

Fig E. Visualisation of the cross-tabulation between the forest loss years in GFC and PRODES.

Table D. Land cover 1–2 years prior to forest loss (NB: only for where land cover data for the relevant time period was available, so the area expressed in Mha does not cover all of the deforestation).

GlobeLand	GFC	PRODES	TerraClass	GFC	PRODES
Forest	58% (1.8 Mha)	71% (1.1 Mha)	Forest	43% (1.2 Mha)	97% (1.0 Mha)
Grassland	25% (0.8 Mha)	19% (0.3 Mha)	Pasture	21% (0.6 Mha)	1% (<0.1 Mha)
Cultivated land	3% (0.1 Mha)	<1%	Annual crops	1% (<0.1 Mha)	<1%
Other	14% (0.4 Mha)	9% (0.1 Mha)	Other	6% (0.2 Mha)	<1%
			Non-forest/2nd veg.	29% (0.9 Mha)	1% (<0.1 Mha)

Intercomparison TerraClass and GlobeLand

Many of the differences between TerraClass and GlobeLand30 lie in TerraClass' Non-forest and Secondary vegetation classes. These do not have a direct correspondence to either of the GlobeLand30 classes, and are not necessarily inconsistencies.

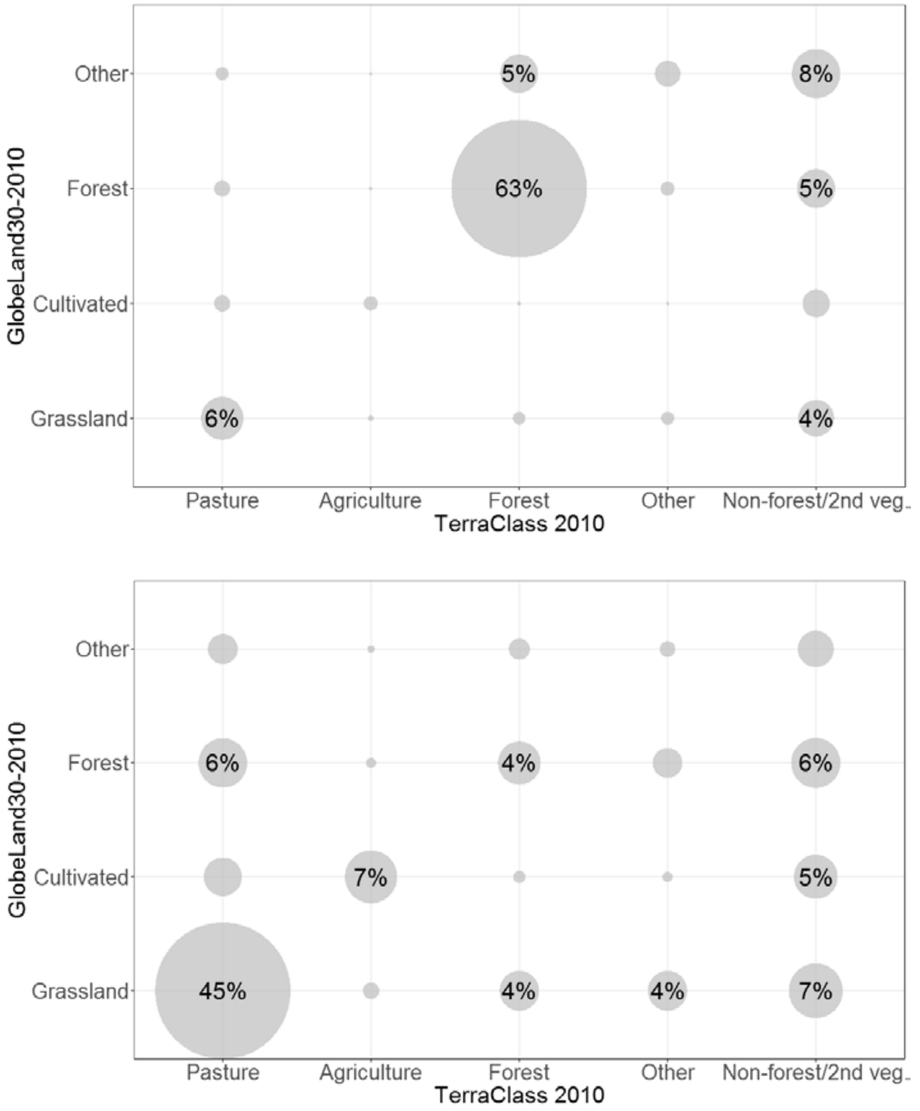

Fig F. Bubble charts of TerraClass 2010 and GlobeLand30-2010 land cover/use classes. Top: for all pixels (except where GFC or PRODES show forest loss in-between their timing). Bottom: following GFC forest loss prior to 2010 (and to timing of land cover data). Percentage labels show the percentage of the total area in each of the combination of classes.

Post-loss forest

Fig G presents the spatial variations in the proportion of forest loss that is followed by forest. In some countries (e.g. Nicaragua, Colombia, and Peru) forest comes up as the dominant post-loss land cover in general, while in others it dominant in certain places only (e.g. Brazil and Paraguay).

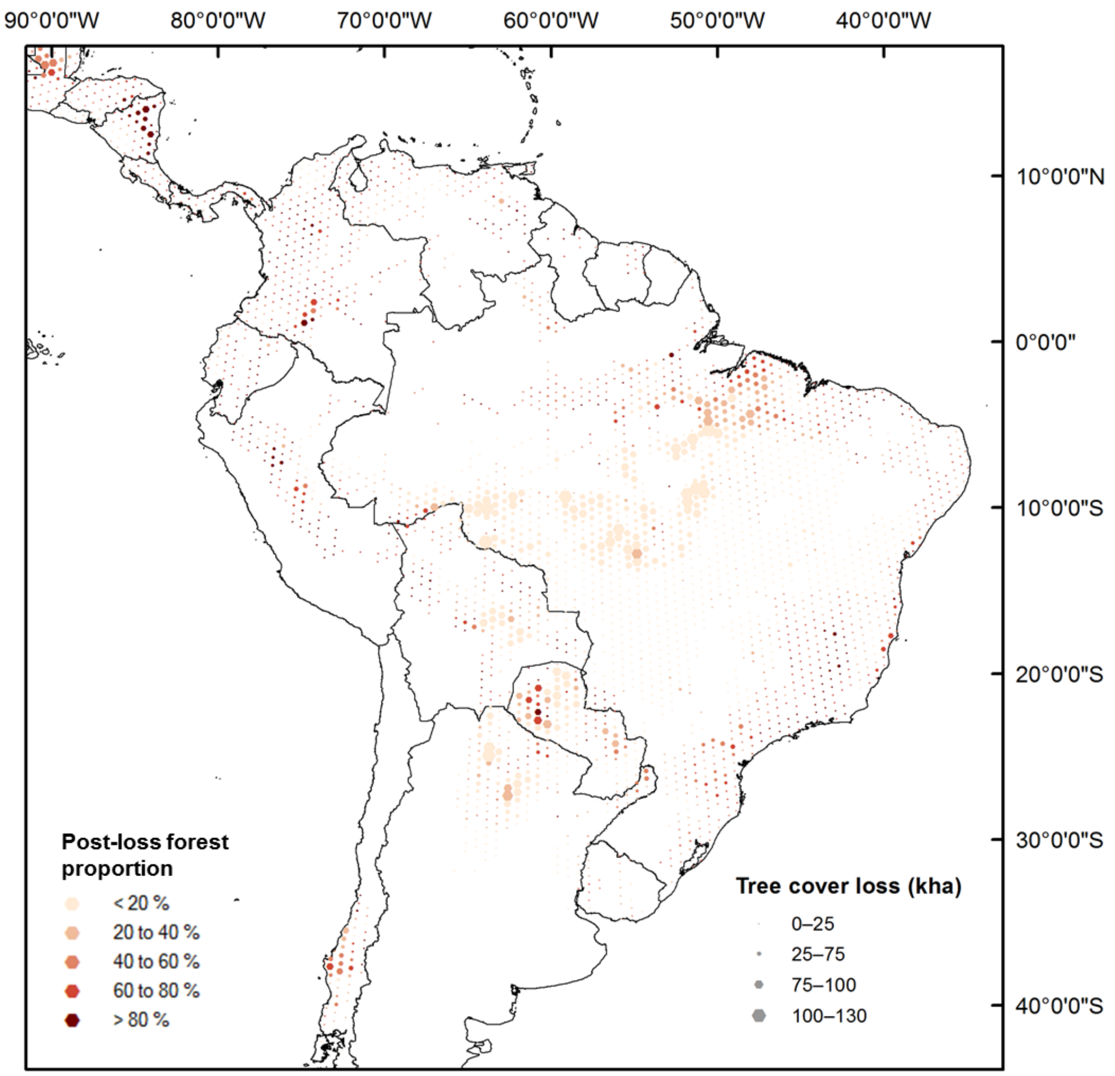

Fig G. Map of post-loss forest.

Boundary pixels

To examine whether pixels near class boundaries (in either of the datasets) are particularly likely to have a large proportion of forest as post-loss land cover, we calculate the proportion of forest loss pixels in each hexagon that are also on the boundary between classes in either of the (GlobeLand30 or GFC) datasets.

To detect class boundaries, we use R raster boundaries ("Rooks case", where boundaries are defined as pixels with more than one class in the four neighbouring pixels; based on the joint raster of GFC forest loss prior to GlobeLand30, resampled with FME to match better the input resolution), and summarised the resulting "boundary raster" per hexagon. We then use the ratio between boundaries and non-boundaries and compare this to the proportion of forest loss pixels followed by forest in the GlobeLand30 data.

If there were no correlation between frequency of boundaries and the share of post-loss forest, we would expect that the range of boundary ratios would be the same between hexagons with low and high proportions of "post-loss forest". However, as seen in a majority of the hexagons with large proportions of "post-loss forest" are also dominated by a large proportion of boundaries (Fig H). (Although there are also several outliers; in these hexagons, other sources of error is likely. This relationship could be used to identify locations of larger errors.) Hexagons with lower "post-loss forest" proportions, exhibit a greater range in boundary proportions. This is even clearer if we exclude hexagons with less than 5 kha forest loss. This is unlike the proportions of Grassland and Cultivated land, which exhibit the reverse pattern (Fig H).

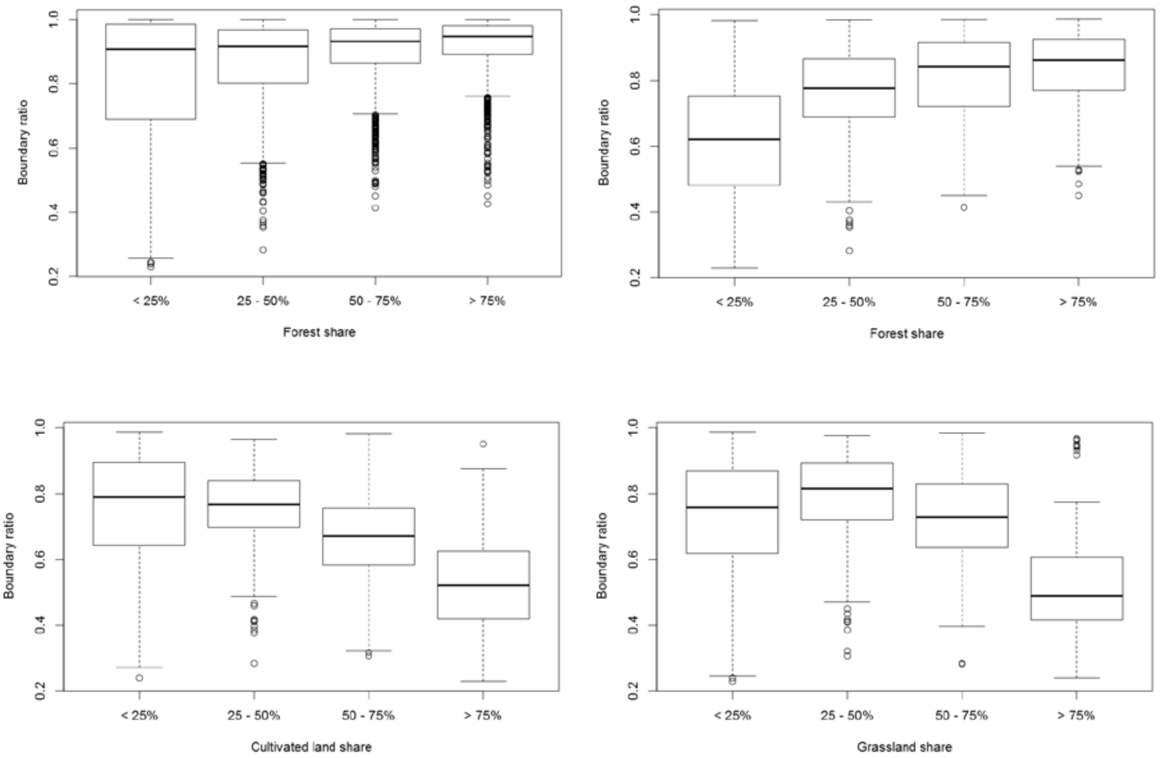

Fig H. Boxplots of boundary ratio depending on share of: forest (for all hexagons, top left, and only for hexagons with at least 5kha forest loss, top right), Cultivated land (bottom left) and Grassland (bottom right) (the latter two also for at least 5 kha forest loss).

A visual inspection of some of the larger areas of post-loss forest classification reveal incorrect classification by GlobeLand30 and false positives by GFC (including some instances which seem to be related to Landsat's SLC OFF). An example of where misclassification seem to be an explanation for post-loss forest are given in Fig I.

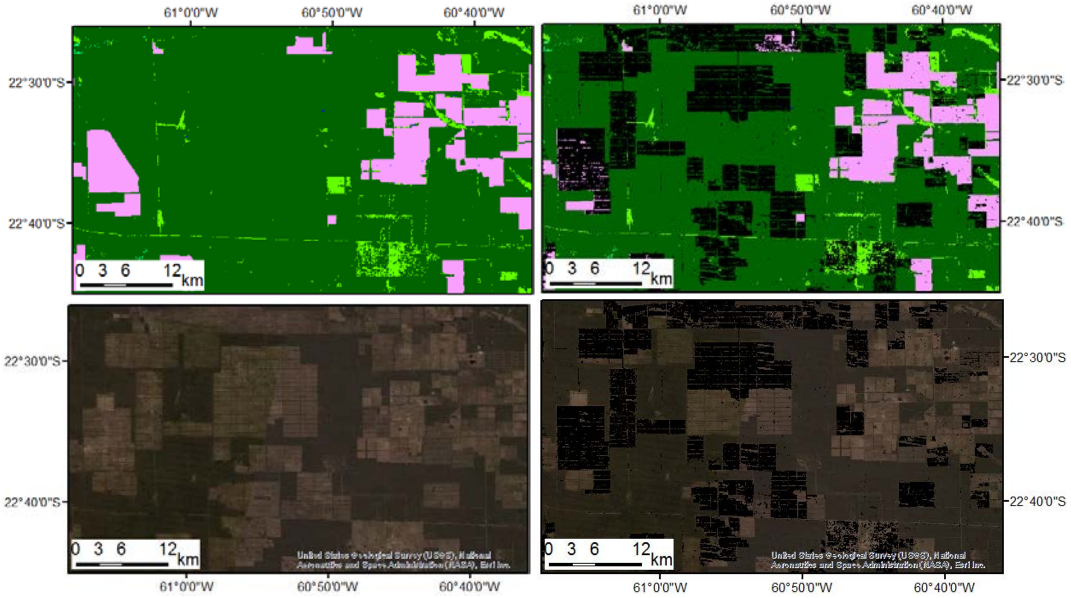

Fig I. Example of likely incorrect forest classification by GlobeLand30 in Boquerón, Paraguay. Top: GlobeLand30 data (based on 2010 Landsat data) with forest shown in dark green. GFC forest loss 2001–2008 shown in black (top right, bottom right). Bottom: same area shown in Landsat Global Land Survey 2010 Enhanced (Landsat TM 5 and 7, SLC-corrected), from United States Geological Survey (USGS), National Aeronautics and Space Administration (NASA), Esri Inc. GlobeLand30 dataset provided by the NGCC (DOI10.11769/GlobeLand30.2010.db) [1, 2], GFC forest loss data source: Hansen/UMD/Google/USGS/NASA.

Another factor is that GlobeLand30's definition of forest is quite broad, and includes sparse woodland with 10–30% canopy cover [1]. For post-loss forest in the BLA, the comparison with TerraClass showed that what is classified by GlobeLand30 as forest (following forest loss), is a mix of a third Non-forest/secondary vegetation, a third Pasture, and a quarter forest (S5 Table).

While one way of dealing with the higher error incidence at class boundaries is to exclude pixels on class boundaries and thus improve the overall accuracy of the remaining dataset (as is done by e.g. Serra et al. [15] and Brovelli et al. [3]), this would likely influence the resulting post-loss land cover, favouring results of large-scale forest loss in areas with homogenous land cover, and underestimate the importance of deforestation drivers that cause of smaller-scale forest loss and/or loss in fragmented landscapes.

References

1. Chen J, Chen J, Liao A, Cao X, Chen L, Chen X, et al. Global land cover mapping at 30 m resolution: A POK-based operational approach. ISPRS J Photogramm Remote Sens. 2015;103:7-27. doi: 10.1016/j.isprsjprs.2014.09.002.
2. National Geomatics Center of China. 30-meter global land cover dataset (GlobeLand30) product description. National High Technology Research and Development Program of China (863 Program), 2014 Project No.: 2009AA122000.
3. Brovelli M, Molinari M, Hussein E, Chen J, Li R. The first comprehensive accuracy assessment of GlobeLand30 at a national level: methodology and results. Remote Sens (Basel). 2015;7(4):4191. PubMed PMID: doi:10.3390/rs70404191.
4. Jokar Arsanjani J, See L, Tayyebi A. Assessing the suitability of GlobeLand30 for mapping land cover in Germany. Int J Digit Earth. 2016;9(9):873-91. doi: 10.1080/17538947.2016.1151956.

5. Jokar Arsanjani J, Tayyebi A, Vaz E. GlobeLand30 as an alternative fine-scale global land cover map: Challenges, possibilities, and implications for developing countries. Habitat Int. 2016;55:25-31. doi: 10.1016/j.habitatint.2016.02.003.
6. Lambert M-J, Waldner F, Defourny P. Cropland mapping over Sahelian and Sudanian agrosystems: a knowledge-based approach using PROBA-V time series at 100-m. Remote Sens (Basel). 2016;8(3):232. PubMed PMID: doi:10.3390/rs8030232.
7. Zhao Y, Gong P, Yu L, Hu L, Li X, Li C, et al. Towards a common validation sample set for global land-cover mapping. Int J Remote Sens. 2014;35(13):4795-814. doi: 10.1080/01431161.2014.930202.
8. Yang Y, Xiao P, Feng X, Li H. Accuracy assessment of seven global land cover datasets over China. ISPRS J Photogramm Remote Sens. 2017;125:156-73. doi: http://dx.doi.org/10.1016/j.isprsjprs.2017.01.016.
9. Hansen MC, Potapov PV, Moore R, Hancher M, Turubanova SA, Tyukavina A, et al. High-resolution global maps of 21st-century forest cover change. Science. 2013;342(6160):850-3. doi: 10.1126/science.1244693.
10. Tyukavina A, Baccini A, Hansen MC, Potapov PV, Stehman SV, Houghton RA, et al. Aboveground carbon loss in natural and managed tropical forests from 2000 to 2012. Environ Res Lett. 2015;10(7):074002.
11. Camara G, Valeriano D, Vianei J, Motta Md, Maurano L. Metodologia para o cálculo da taxa anual de desmatamento na Amazônia Legal [Methodology for the calculation of annual deforestation rates in the Legal Amazon]. São José dos Campos, Brazil: Instituto Nacional de Pesquisas Espaciais (INPE), 2013.
12. Almeida CAd, Coutinho AC, Esquerdo JCDM, Adami M, Venturieri A, Diniz CG, et al. High spatial resolution land use and land cover mapping of the Brazilian Legal Amazon in 2008 using Landsat-5/TM and MODIS data. Acta Amazon. 2016;46:291-302.
13. Richards P, Arima E, VanWey L, Cohn A, Bhattarai N. "Are Brazil's deforesters avoiding detection?". Conserv Lett. 2016:n/a-n/a. doi: 10.1111/conl.12310.
14. Fanin T, van der Werf GR. Relationships between burned area, forest cover loss, and land cover change in the Brazilian Amazon based on satellite data. Biogeosciences. 2015;12(20):6033-43. doi: 10.5194/bg-12-6033-2015.
15. Serra P, Pons X, Saurí D. Post-classification change detection with data from different sensors: Some accuracy considerations. Int J Remote Sens. 2003;24(16):3311-40. doi: 10.1080/0143116021000021189.
