# Supplementary material for: Combining global land cover datasets to quantify agricultural expansion into forests in Latin America: Limitations and challenges
Source: PLoS One. 2017 Jul 13;12(7):e0181202. doi: 10.1371/journal.pone.0181202 (PMC5509295; doi:10.1371/journal.pone.0181202)
Supplement: S2 Appendix — (PDF) [file pone.0181202.s007.pdf]

S2 Appendix. Impact of lag time on post-loss land cover.

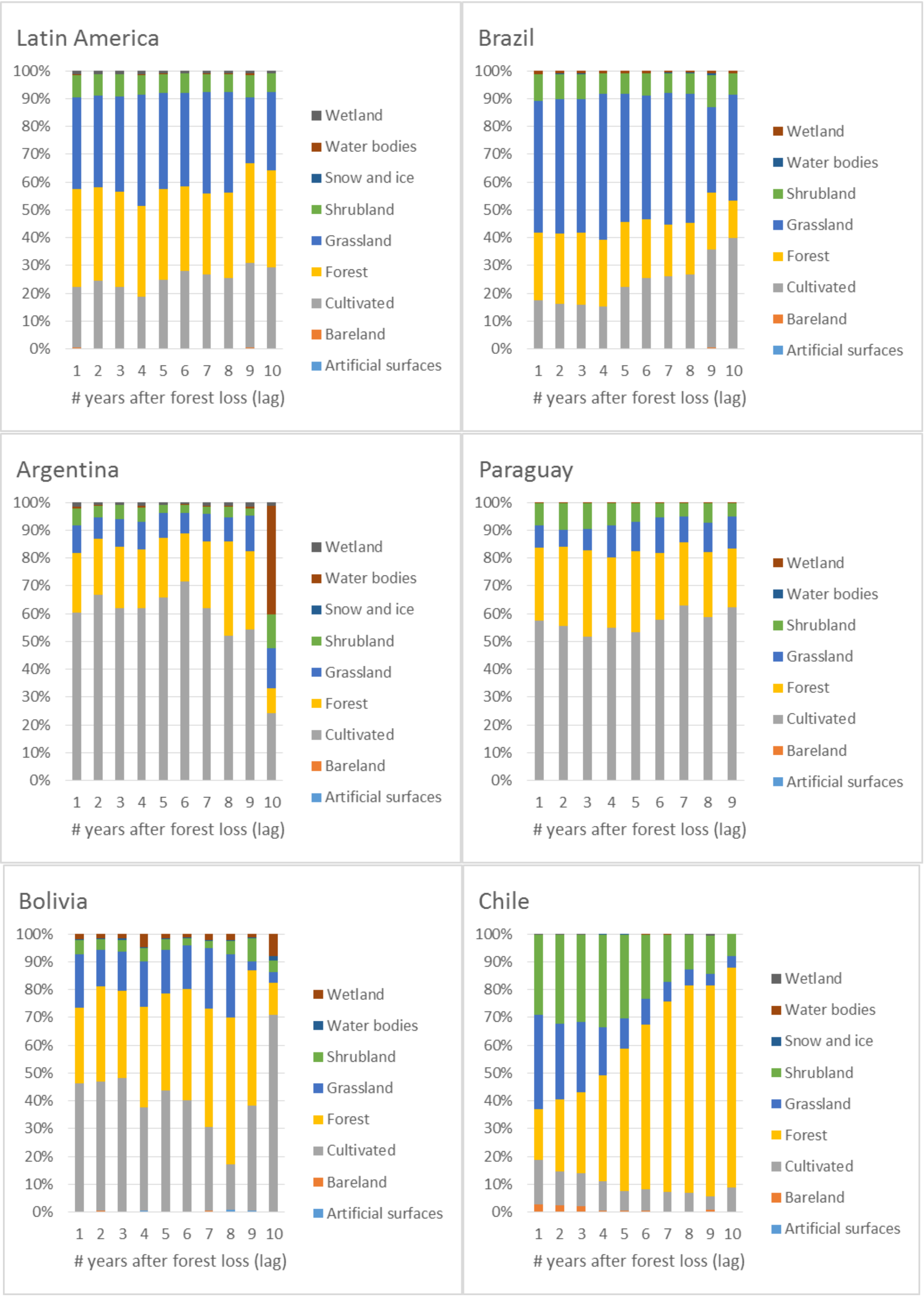

Figure A. Share of GlobeLand30 land cover depending on lag time (number of years) between forest loss and land cover determination.

To determine the number of years between forest loss and the post-loss land cover to use, we experimented with varying the interval for different countries, and found that varying the interval used between the forest loss event and the post-loss land cover did not particularly alter the distribution of post-loss land cover types, for the countries with the most forest loss (as well as for Latin America seen as a whole). However, as very few pixels fall into intervals of 10 years or more, summarised results may seem erratic for longer lags, as they will be determined on the basis of only a few pixels (there is, however, no indication from previous years that would be a basis for excluding these pixels from the analysis).
