# Supplementary material for: Combining global land cover datasets to quantify agricultural expansion into forests in Latin America: Limitations and challenges
Source: PLoS One. 2017 Jul 13;12(7):e0181202. doi: 10.1371/journal.pone.0181202 (PMC5509295; doi:10.1371/journal.pone.0181202)
Supplement: S3 Appendix — Maps over study area showing biomes and ecoregions from Terrestrial Ecoregions of the World. (PDF) [file pone.0181202.s008.pdf]

S3 Appendix. Post-loss GlobeLand30-2010 land cover per ecoregion, and study area maps of Terrestrial Ecoregions of the World.

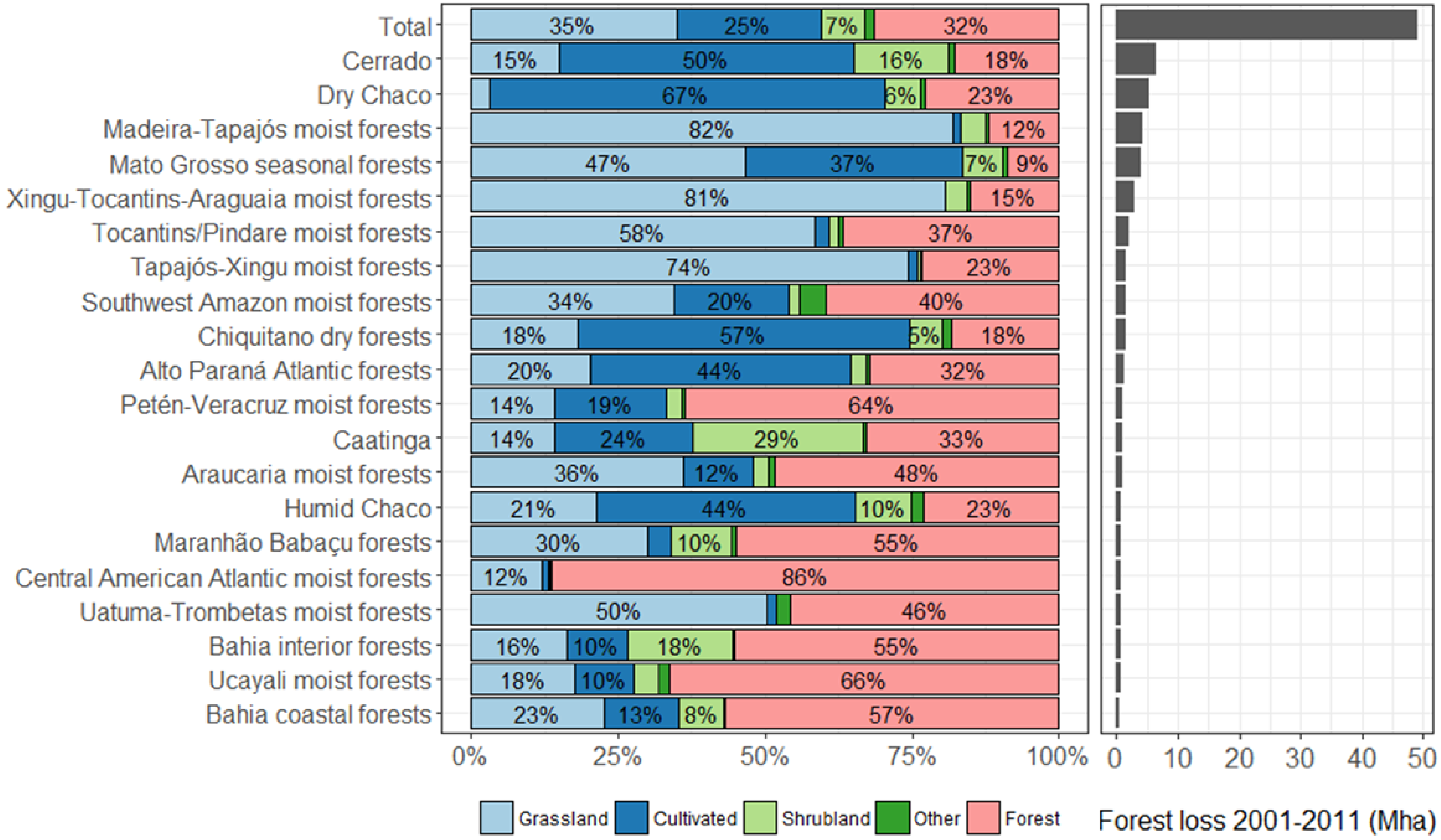

Figure A. Forest loss 2001–2011 and post-loss land cover per ecoregion (A) Proportion of GlobeLand-30 land cover types following GFC forest loss. (B) Tree cover loss 2001–2011 per ecoregion, detected by GFC. Ecoregion boundaries from Terrestrial Ecoregions of the World (Olson et al., 2001).

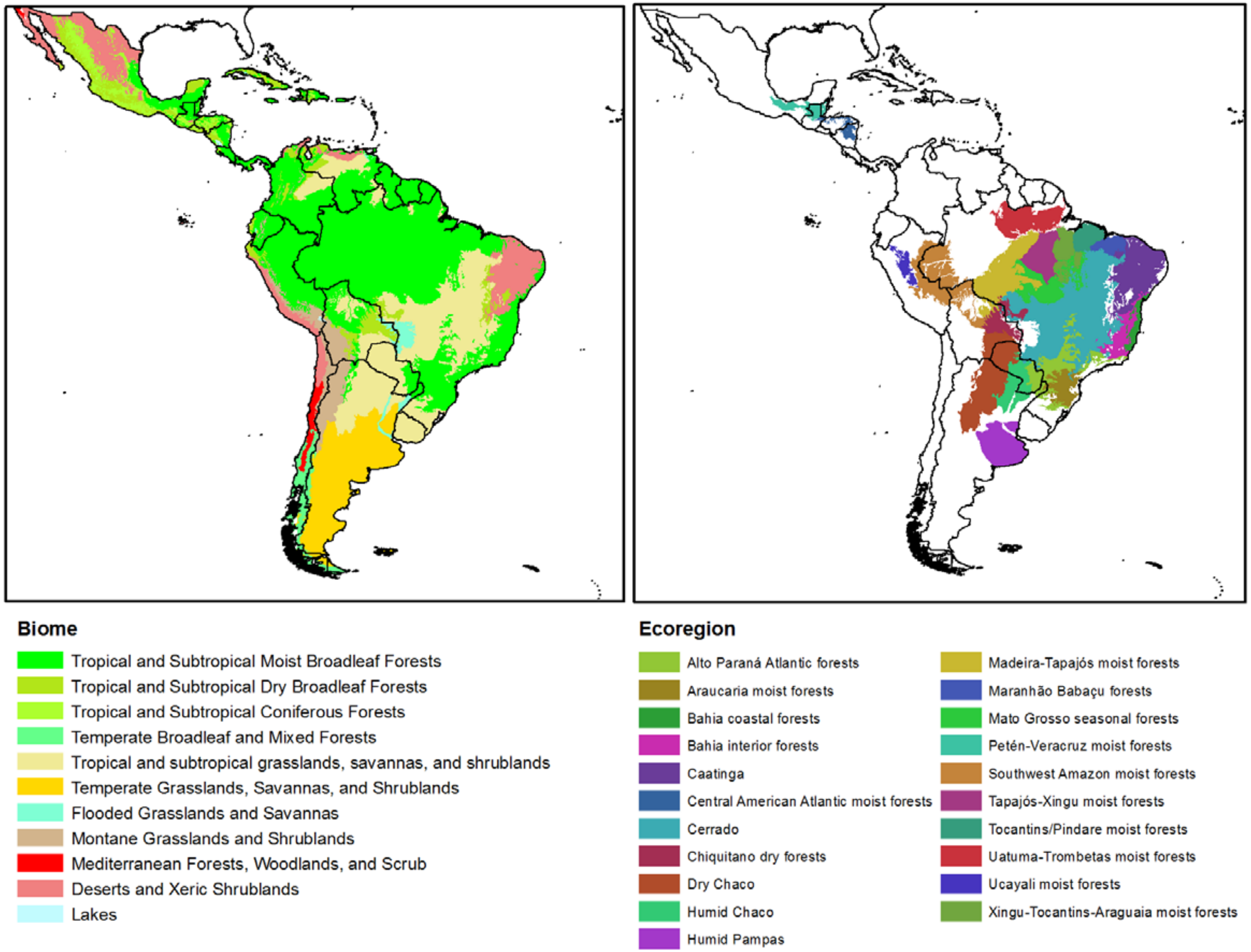

Figure B. Map of selected biomes and ecoregions from Olson et al. [1]. Only the ecoregions with the most forest loss 2001 – 2011 are shown. Map dataset provided by Terrestrial Ecoregions of the World (Available from: https://www.worldwildlife.org/publications/terrestrial-ecoregions-of-the-world).

1. Olson DM, Dinerstein E, Wikramanayake ED, Burgess ND, Powell GVN, Underwood EC, et al. Terrestrial Ecoregions of the World: A New Map of Life on Earth. Bioscience. 2001;51(11):933-8. doi: 10.1641/0006-3568(2001)051[0933:TEOTWA]2.0.CO;2.
