# Supplementary figures and images for: Combining global land cover datasets to quantify agricultural expansion into forests in Latin America: Limitations and challenges
Source: PLoS One. 2017 Jul 13;12(7):e0181202. doi: 10.1371/journal.pone.0181202 (PMC5509295; doi:10.1371/journal.pone.0181202)

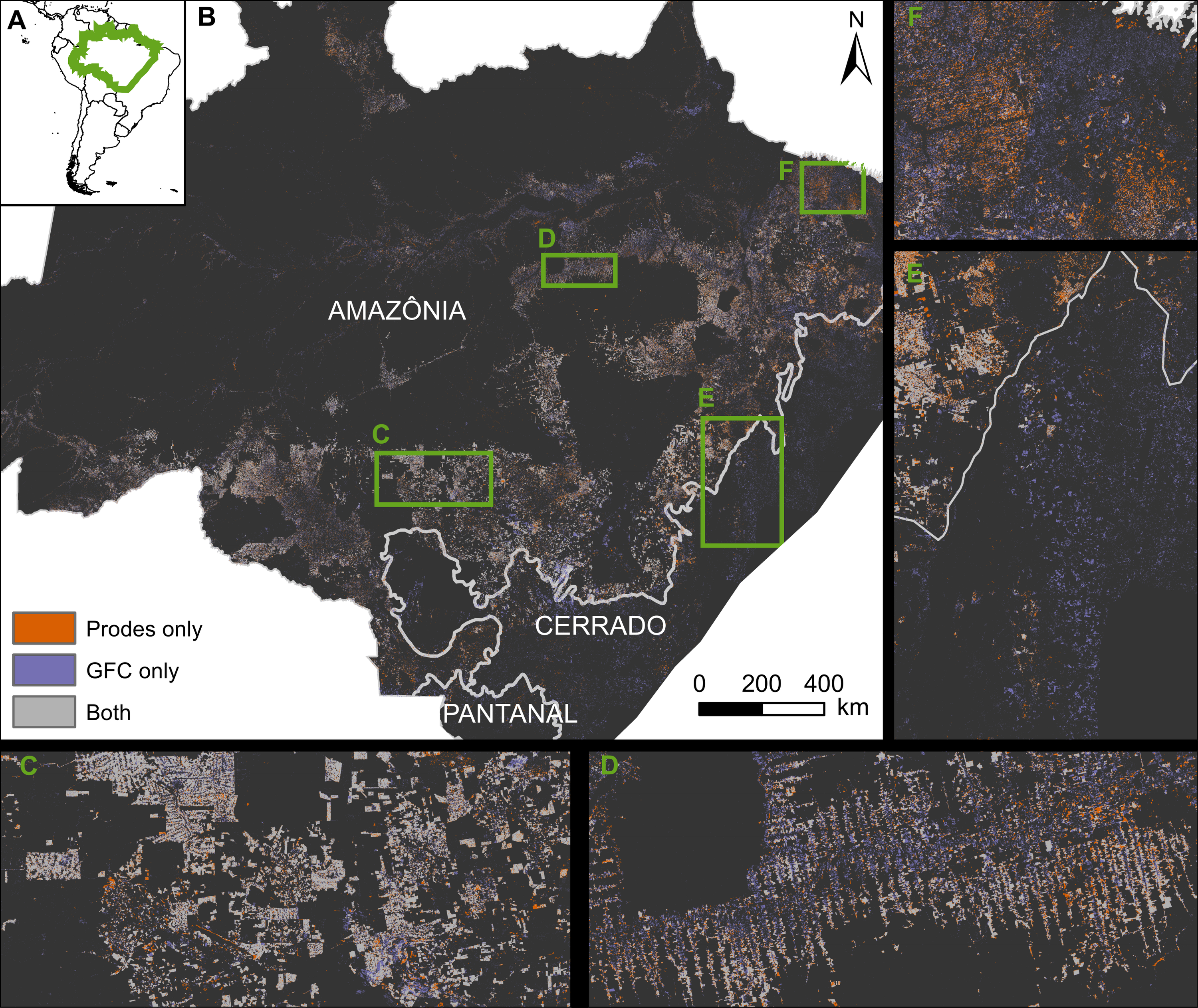

Supplement: S1 Fig — PRODES deforestation and GFC tree cover loss detected during the years 2001–2014 in their common extent in the Brazilian Legal Amazon, as well as some close-ups. Grey shows where both datasets report loss at some point during the time period (differences in timing of the loss event are not distinguished for in this figure). (TIF) [file pone.0181202.s009.tif]

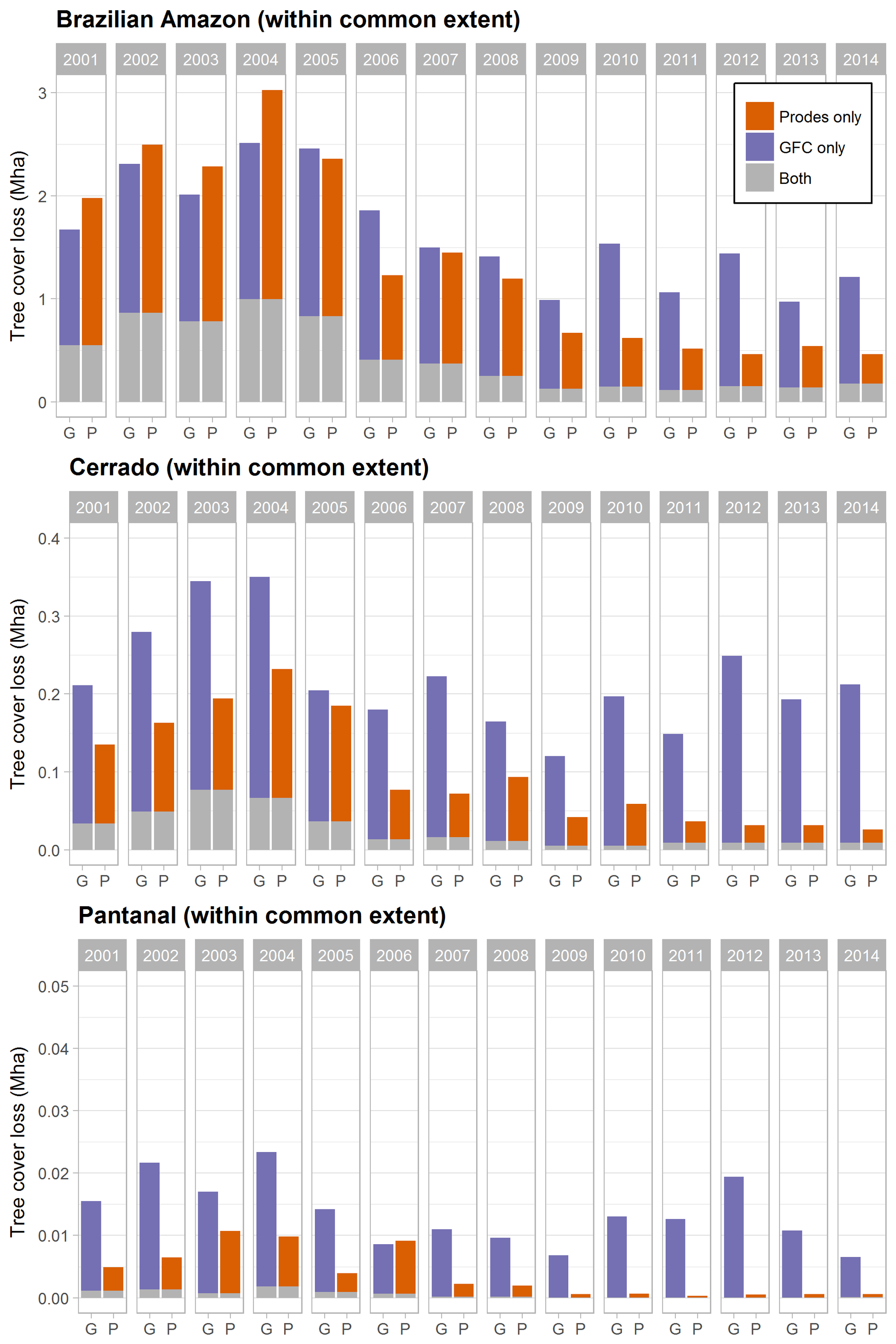

Supplement: S2 Fig — For areas where both datasets were available (i.e. mainly the Amazon biome). Note change in scale of the y-axis. (TIF) [file pone.0181202.s010.tif]
